# MFSD7c functions as a transporter of choline at the blood–brain barrier

**DOI:** 10.1038/s41422-023-00923-y

**Published:** 2024-02-02

**Authors:** Xuan Thi Anh Nguyen, Thanh Nha Uyen Le, Toan Q. Nguyen, Hoa Thi Thuy Ha, Anna Artati, Nancy C. P. Leong, Dat T. Nguyen, Pei Yen Lim, Adelia Vicanatalita Susanto, Qianhui Huang, Ling Fam, Lo Ngah Leong, Isabelle Bonne, Angela Lee, Jorge L. Granadillo, Catherine Gooch, Dejie Yu, Hua Huang, Tuck Wah Soong, Matthew Wook Chang, Markus R. Wenk, Jerzy Adamski, Amaury Cazenave-Gassiot, Long N. Nguyen

**Affiliations:** 1 Department of Biochemistry, Yong Loo Lin School of Medicine, National University of Singapore, Singapore, Singapore; 2 Metabolomics and Proteomics Core, Helmholtz Center Munich, German Research Center for Environmental Health, Neuherberg, Germany; 3 Singapore Lipidomics Incubator (SLING), Life Sciences Institute, National University of Singapore, Singapore, Singapore; 4 NUS Synthetic Biology for Clinical and Technological Innovation (SynCTI), National University of Singapore, Singapore, Singapore; 5 Synthetic Biology Translational Research Programme, Yong Loo Lin School of Medicine, National University of Singapore, Singapore, Singapore; 6 Electron Microscopy Unit, Yong Loo Lin School of Medicine, National University of Singapore, Singapore, Singapore; 7 Department of Microbiology and Immunology, Immunology Translational Research Programme, Yong Loo Lin School of Medicine, National University of Singapore, Singapore, Singapore; 8 Life Sciences Institute, Immunology Programme, National University of Singapore, Singapore, Singapore; 9 Division of Genetics and Genomic Medicine, Department of Pediatrics, Washington University in St Louis, Saint Louis, MO USA; 10 Electrophysiology Core Facility, Yong Loo Lin School of Medicine, National University of Singapore, Singapore, Singapore; 11 Department of Physiology, Yong Loo Lin School of Medicine, National University of Singapore, Singapore, Singapore; 12 Healthy Longevity Translational Research Program, Yong Loo Lin School of Medicine, National University of Singapore, Singapore, Singapore; 13 Cardiovascular Diseases Program, National University of Singapore, Singapore, Singapore; 14 Institute of Experimental Genetics, Helmholtz Zentrum München, German Research Center for Environmental Health, Neuherberg, Germany; 15 Institute of Biochemistry, Faculty of Medicine, University of Ljubljana, Ljubljana, Slovenia; 16 Immunology Translational Research Program, Yong Loo Lin School of Medicine, National University of Singapore, Singapore, Singapore; 17 Present Address: Hudson Institute of Medical Research, Clayton, VIC Australia

**Keywords:** Cell biology, Protein transport

## Abstract

Mutations in the orphan transporter *MFSD7c* (also known as *Flvcr2*), are linked to Fowler syndrome. Here, we used *Mfsd7c* knockout (*Mfsd7c*^–/–^) mice and cell-based assays to reveal that MFSD7c is a choline transporter at the blood–brain barrier (BBB). We performed comprehensive metabolomics analysis and detected differential changes of metabolites in the brains and livers of *Mfsd7c*^–/–^embryos. Particularly, we found that choline-related metabolites were altered in the brains but not in the livers of *Mfsd7c*^–/–^ embryos. Thus, we hypothesized that MFSD7c regulates the level of choline in the brain. Indeed, expression of human *MFSD7c* in cells significantly increased choline uptake. Interestingly, we showed that choline uptake by MFSD7c is greatly increased by choline-metabolizing enzymes, leading us to demonstrate that MFSD7c is a facilitative transporter of choline. Furthermore, single-cell patch clamp analysis showed that the import of choline by MFSD7c is electrogenic. Choline transport function of MFSD7c was shown to be conserved in vertebrates, but not in yeasts. We demonstrated that human MFSD7c is a functional ortholog of HNM1, the yeast choline importer. We also showed that several missense mutations identified in patients exhibiting Fowler syndrome had abolished or reduced choline transport activity. Mice lacking *Mfsd7c* in endothelial cells of the central nervous system suppressed the import of exogenous choline from blood but unexpectedly had increased choline levels in the brain. Stable-isotope tracing study revealed that MFSD7c was required for exporting choline derived from lysophosphatidylcholine in the brain. Collectively, our work identifies MFSD7c as a choline exporter at the BBB and provides a foundation for future work to reveal the disease mechanisms of Fowler syndrome.

## Introduction

Missense mutations in *MFSD7c* (also known as *FLVCR2*) have been associated with Fowler syndrome.^1^ The major clinical symptoms reported in these patients are the severe dilation of cerebral blood vessels and mild microcephaly. Most severe cases result in embryonic death, but some patients have survived after birth.^1–6^ Although the cause of Fowler syndrome is unclear, it is linked to the loss of functions of MFSD7c, an orphan transporter. To gain a better understanding of the disease mechanisms, the molecular role of MFSD7c must be elucidated. We recently reported that mice with a global deletion of *Mfsd7c* had late gestation lethality with severe dilation of central nervous system (CNS) vasculature.^2^ Specific deletion of *Mfsd7c* in endothelial cells also resulted in similar phenotypes, indicative of its critical roles in the blood vessels.^7^ *Mfsd7c* knockout (KO) embryos exhibited multiple neurological phenotypes that are probably linked to primary defects of the CNS vasculature. These phenotypes resemble the glomeruloid structures in the CNS blood vessels of patients with Fowler syndrome.^2,7^

MFSD7c belongs to the Major Facilitator Superfamily (MFS) of transporters which facilitate the movement of small molecules through cell membranes.^8^ MFSD7c was reported to exert heme import activity.^9^ However, this observation has not been confirmed in the literature. Recently, it has been shown that MFSD7c is involved in thermogenesis in response to heme. Besides its localization in the mitochondria, this study and our results also showed that MFSD7c was expressed in the plasma membrane.^2,10^ Furthermore, we and others showed that MFSD7c was expressed in endothelial cells of the CNS vasculature.^2,7^ These results suggest that MFSD7c is a membrane transporter for small molecules at the blood–brain barrier (BBB). Utilizing mouse KO models coupled with comprehensive metabolomics analyses, we revealed the specific changes in metabolites due to the loss of *Mfsd7c*. These results guide us to demonstrate that MFSD7c is a transporter for choline. We show that MFSD7c facilitates choline transport via the plasma membrane in a concentration-dependent manner. Unexpectedly, we find that lack of MFSD7c at the BBB causes choline accumulation in the brain. A stable isotopic tracing study reveals that choline from plasma lysophosphatidylcholine (LPC) is delivered to the brain. The excessive amount of choline is exported out of the brain via MFSD7c. The current work identifies MFSD7c as a choline transporter and provides a foundation for future studies to reveal the disease mechanisms of Fowler syndrome. Additionally, the identification of MFSD7c as a choline transporter at the BBB lays the groundwork for future research to reveal the metabolic fates of choline in the brain.

## Results

### Lack of *Mfsd7c* affects choline levels in the brain

**Fig. 1:**
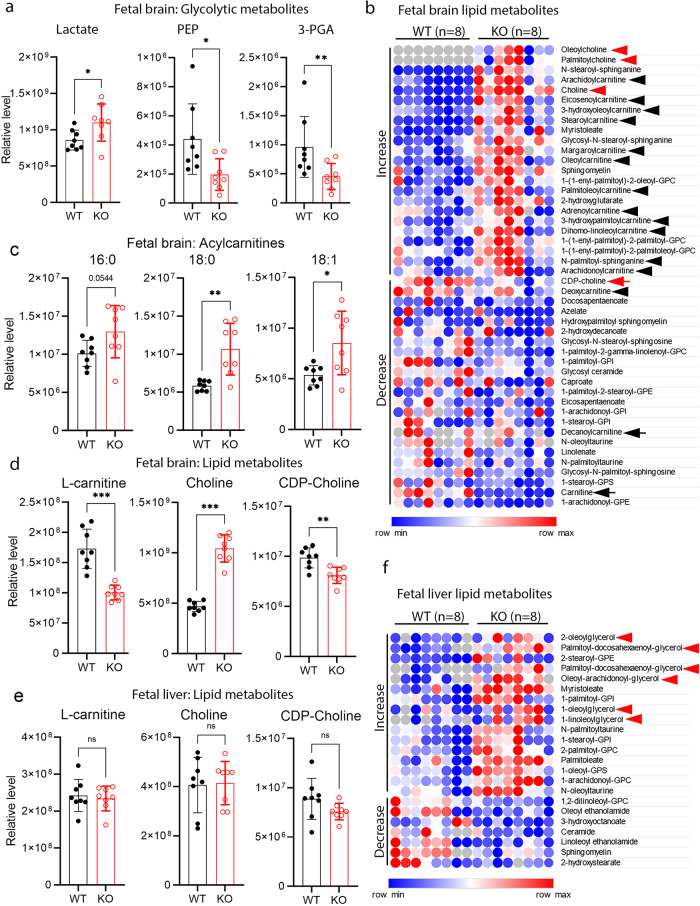
Comprehensive metabolomics analysis identifies changes in the levels of choline and choline metabolites in the brain of *Mfsd7c* KO embryos. **a** Increased levels of glycolytic metabolite lactate and decreased levels of mitochondrial metabolites in *Mfsd7c* KO brain. **b** Heatmap of metabolites with differential changes in the brains of *Mfsd7c* KO compared to that of WT embryos. **c** Increased levels of acylcarnitines in the brains of *Mfsd7c* KO embryos. **d** Increased levels of choline and decreased levels of CDP-choline and L-carnitine in the brains of *Mfsd7c* KO embryos. **e** Levels of L-carnitine, choline, and CDP-choline were unchanged in the fetal livers of WT and *Mfsd7c* KO embryos. **f** Heatmap of neutral lipid species that are differentially changed in the fetal livers of *Mfsd7c* KOs compared to that of WT embryos. The results showed the accumulation of neutral lipids in the fetal livers of the *Mfsd7c* KO embryos. The brains and livers were collected at E14.5. *n* = 8 per genotype. ***P < 0.001, **P < 0.01, *P < 0.05; non-parametric *t*-test (Mann–Whitney test); ns, not significant. Data are mean ± SD; Each dot represents one mouse. Gray circles are not detectable metabolites.

Deletion of *Mfsd7c* resulted in late gestation lethality in mice.^2,7^ Because MFSD7c is predicted to be a membrane transporter, its deletion is anticipated to cause insufficient transport of essential nutrients in and/or out of the brain. To deorphanize the physiological ligands for MFSD7c, we performed a comprehensive analysis of metabolites in the brains of wild-type (WT) and global *Mfsd7c* KO (*Mfsd7c*^–/–^) embryos at gestation day 14.5 (E14.5), a time point when KO embryos already exhibited vascular growth defects.^2^ This analysis encompassed more than 520 metabolites from metabolic pathways for carbohydrates, lipids, amino acids, and cofactors (Supplementary information, Tables S1–S10). First, we found that the levels of glycolytic metabolites such as lactate were significantly increased, whereas the levels of 3-phosphoglycerate and phosphoenolpyruvate were reduced (Fig. 1a). These metabolic changes are congruent with the increased glycolysis and possibly reduced mitochondrial activity as a consequence of hypoxia that occurs in the brain of *Mfsd7c*^–/–^ embryos.^2,7^ MFSD7c was shown to regulate heme transport.^10^ However, in our data, heme and bilirubin levels in fetal brains were comparable between *Mfsd7c*^*–/–*^ and WT embryos (Supplementary information, Fig. S1a, b). Second, we detected increased levels of several long-chain acylcarnitines (Fig. 1b, c; black arrowheads), whereas the levels of L-carnitine were significantly reduced in the brain of *Mfsd7c*^*–/–*^ embryos (Fig. 1d). Nevertheless, we ruled out that MFSD7c transports these acylcarnitines by performing the import assay with palmitoyl-carnitine (C16:0 carnitine) and octanoyl-carnitine (C8:0 carnitine) (Supplementary information, Fig. S1c, d). Again, these changes might reflect the defects of mitochondrial activity due to hypoxia in the brain of KO embryos. Indeed, mitochondrial morphology and the expression level of several mitochondrial markers were reduced in the brain of *Mfsd7c*^–/–^ embryos (Supplementary information, Fig. S2). Interestingly, we noted increased levels of choline, palmitoylcholine, and oleoylcholine in the brains of *Mfsd7c*^–/–^ embryos relative to WT embryos (Fig. 1b; red arrowheads). In contrast, the levels of CDP-choline were reduced in *Mfsd7c*^–/–^ embryos (Fig. 1d). These changes in choline metabolites were specific to the brain as their levels were unaltered in the livers from *Mfsd7c*^–/–^ embryos (Fig. 1e). Instead, we detected a striking increase in the levels of several neutral lipids such as monoacylglycerols and diacylglycerols in the livers of *Mfsd7c*^–/–^ embryos (Fig. 1f, arrowheads). Deficiency of choline has been linked to mitochondrial defects.^11–14^ These differential changes in the levels of choline and choline metabolites in the brain and the accumulation of neutral lipids in the livers of *Mfsd7c*^–/–^ embryos suggest that MFSD7c may be involved in choline metabolism.

### MFSD7c mediates the transport of choline

**Fig. 2:**
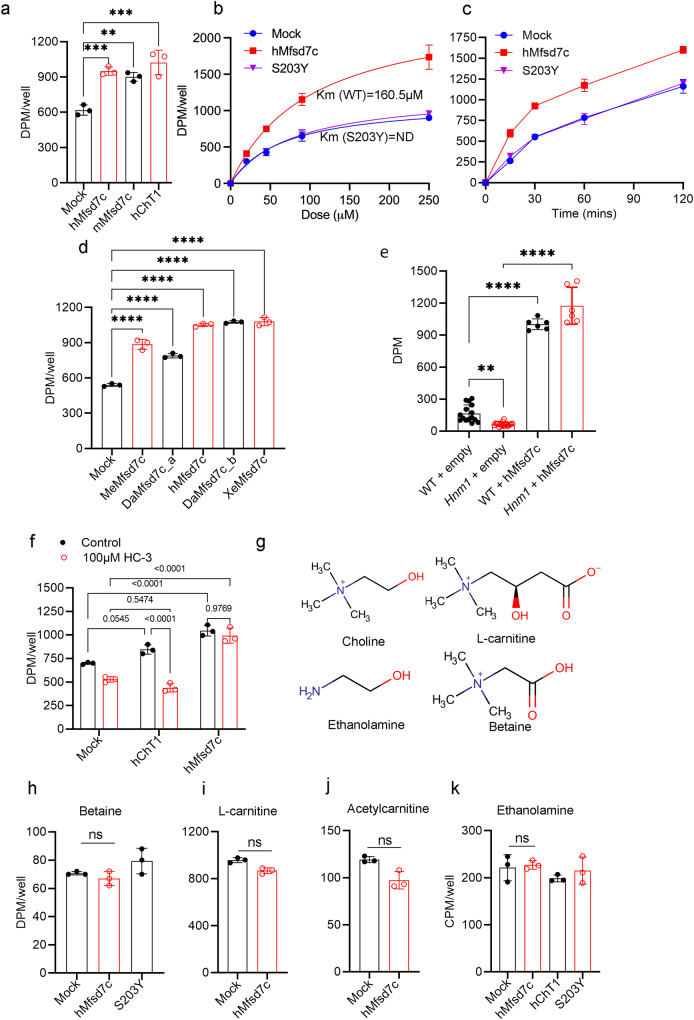
Expression of MFSD7c increases choline uptake. **a** Overexpression of *mMfsd7c* and *hMfsd7c* increases choline uptake in HEK293 cells. CHT1 (SLC5a7), a neuronal choline transporter, was included as a positive control. **b** Dose curve for choline uptake from mock, hMfsd7c, and S203Y mutant. **c** Time course for choline uptake from mock, hMfsd7c, and S203Y mutant. Experiments were performed at least twice in triplicate. **d** Transport activity of mock, *MeMfsd7c*, zebrafish *Mfsd7c* isoform a (DaMfsd7c_a), *hMfsd7c*, zebrafish *Mfsd7c* isoform b (DaMfsd7c_b), and *XeMfsd7c*. **e** Complementation assays of yeast *S. cerevisiae* choline transporter *HNM1* KO cells with hMfsd7c. Transport activity of WT *S. cerevisiae* strain and *Hnm1* mutant strain with and without overexpression of hMfsd7c. The results show that expression of hMfsd7c overcomes the loss of choline transport activity in *HNM1* mutant yeast cells. **f** Choline transport activity by CHT1 and hMfsd7c in the presence of HC-3, a competitive inhibitor of CHT1. **g** Structures of choline, betaine, L-carnitine, and ethanolamine. **h**–**k** Uptake assays of hMfsd7c, S203Y, and/or CHT1 with betaine, L-carnitine, acetyl-carnitine, and ethanolamine. Each symbol represents one biological replicate. All of these experiments were repeated at least twice in triplicate. Data are expressed as mean ± SD. ****P < 0.0001, ***P < 0.001, **P < 0.01; ns, not significant. One-way ANOVA for **a**, **d**, **e**, **f**, **h**, and **k**; two-way ANOVA for **b** and **c**; *t*-test for **i** and **j**.

The accumulation of choline and decrease in CDP-choline levels in the brains of *Mfsd7c*^–/–^ embryos suggest that MFSD7c is important for the regulation of choline levels. Thus, we tested whether MFSD7c regulates choline levels by a transport mechanism. We overexpressed human (*hMFSD7c*) or mouse *Mfsd7c* (*mMfsd7c*) in HEK293 cells and utilized radioactive [^3^H]-choline for transport assays. Interestingly, overexpression of hMFSD7c or mMFSD7c led to 1.5–2-fold increase of intracellular levels of radioactive signals, implicating that MFSD7c mediates the import of choline (Fig. 2a). We validated our choline transport assay by including neuronal choline transporter CHT1 (also known as SLC5a7) as a positive control (Fig. 2a). The import of choline mediated by hMFSD7c was increased with increasing levels of choline in medium and with increased time (Fig. 2b, c). To reaffirm that expression of MFSD7c is necessary for choline import to the cells, we included the missense mutation S203Y in our transport assays. In comparison to WT MFSD7c, the S203Y mutant exhibited abolished choline transport activity when incubated with the indicated concentrations of choline (Fig. 2b, c). The reduced import of choline of the mutant was not due to reduced protein expression levels nor defective localization in the plasma membrane (Supplementary information, Fig. S3a, b). MFSD7c is conserved from fish to humans, but not in yeasts (Supplementary information, Fig. S3c). We tested the choline import function for several *MFSD7c* orthologs including the ones from zebrafish (*DaMfsd7c_a* and *DaMfsd7c_b* isoforms), medaka fish (*MeMfsd7c*), and frog (*XeMfsd7c*). Consistently, these MFSD7c orthologs also exhibited choline transport activity (Fig. 2d). In the yeast *S. cerevisiae*, HNM1 was shown to import choline as well as L-carnitine.^15^ We performed complementation assays of *hMFSD7c* in *Hnm1* KO *S. cerevisiae*. First, we showed that *Hnm1* KO cells exhibited reduced choline uptake compared to WT yeast cells (Fig. 2e). Second, when WT yeast cells and *Hnm1* mutant yeast cells were overexpressed with hMFSD7c, they both exhibited significantly increased choline uptake compared to parental cells (Fig. 2e; Supplementary information, Fig. S4a, b). These results indicate that *hMFSD7c* can rescue the loss of yeast *Hnm1* and reaffirm that MFSD7c is a choline transporter. Choline transport by neuronal choline transporter CHT1 is inhibited by hemicholinium-3 (HC-3), a competitive inhibitor of CHT1.^16,17^ HC-3 inhibited CHT1, but not MFSD7c, suggesting that these two choline transporters exert different transport properties (Fig. 2f). SLC44a1 and SLC44a2 have been reported to be choline transporters. However, we were unable to show choline import activity for these transporters under the tested conditions (Supplementary information, Fig. S4c, d). MFSD7c preferred choline as a substrate as it did not transport betaine, a choline metabolite that is also found in the brain (Fig. 2g, h). Similarly, Mfsd7c did not transport L-carnitine, acetyl-carnitine, ethanolamine, and acetylcholine under the tested conditions (Fig. 2i–k; Supplementary information, Fig. S4e). Together, our results reveal that MFSD7c is a conserved transporter for choline in vertebrates.

### MFSD7c functions as a facilitative transporter for choline

**Fig. 3:**
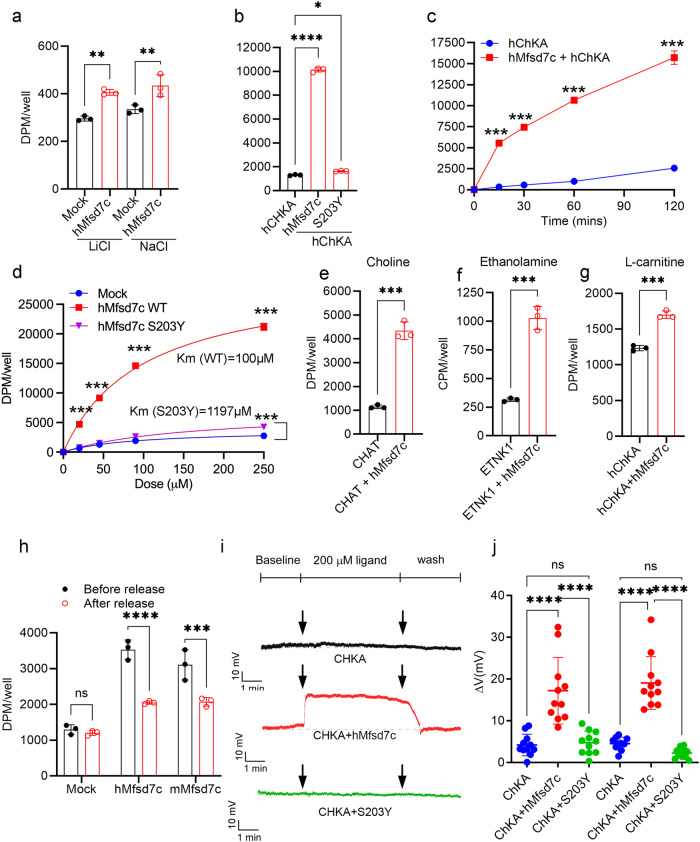
MFSD7c exhibits properties of a facilitative transporter for choline. **a** Transport activity of hMfsd7c is not sodium-dependent. **b** Import of choline by hMfsd7c is strongly increased by co-expression of hChKA. **c** Time course of choline uptake by hMfsd7c when co-expressed with hChKA. **d** Dose curve of choline uptake by hMfsd7c and S203Y mutant when co-expressed with hChKA. Data are expressed as mean ± SD. These experiments were repeated at least twice in triplicate. ***P < 0.0001, **P < 0.01, *P < 0.05. One-way ANOVA: (**a**) and (**b**). Two-way ANOVA: (**c**) and (**d**). **e** Import of choline by hMfsd7c is also significantly increased with co-expression of CHAT. **f** Co-expression of hMfsd7c with ETNK1 increases the uptake of ethanolamine. **g** Co-expression of hMfsd7c with hChKA slightly increased uptake of L-carnitine. **h** hMfsd7c or mMfsd7c mediates the release of choline from cells. Before release: the levels of choline in the cells; After release: the remaining levels of choline in the cells after 30-min incubation with plain DMEM. There was a reduction of intracellular choline due to the release into the medium. Data are expressed as mean ± SD. These experiments were repeated at least twice in triplicate. ****P < 0.0001, ***P < 0.001, *t*-test: (**e**–**g**); one-way ANOVA: (**h**). **i** Uptake of choline by hMfsd7c increases membrane potential in HEK293 cells. Representative exemplar traces of HEK293 cells co-transfected with empty pIRES2-eGFP plasmid and hChKA plasmid (top), hChKA with hMfsd7c (middle), and hChKA with S203Y (bottom). The effect of 200 µM choline on the resting membrane potential changes was induced by the active import of choline by hMfsd7c, but not mutant S203Y. **j** Quantification of resting membrane potential changes induced with 100 and 200 µM choline. Data are expressed as mean ± SD. ****P < 0.0001; ns, not significant; one-way ANOVA.

We noted that the transport activity of MFSD7c was slightly increasing over time (Fig. 2c), suggesting that intracellular levels of choline might affect the influx of choline. Thus, we characterized its transport properties by testing whether MFSD7c utilizes cations for transport activity. Replacement of sodium with lithium did not affect choline import by MFSD7c (Fig. 3a; Supplementary information, Fig. S5a). Next, we tested whether lowering intracellular choline levels could increase the influx of choline via MFSD7c. To test this hypothesis, we co-expressed *hMfsd7c* with human choline kinase A (*hChKA*), which phosphorylates choline to phosphorylcholine, thus lowering the intracellular concentration of choline. Remarkably, co-expression of *hMfsd7c* with *hChKA* increased choline uptake by approximately 9-fold (Fig. 3b), whereas sole expression of *hMfsd7c* increased choline uptake by 1.5–2-fold (Fig. 2a). In the presence of *hChKA*, the import of choline was significantly increased over time (Fig. 3c). Co-expression of *hChKA* with *hMfsd7c* also greatly enhanced choline uptake in a dose-dependent manner (Fig. 3d; Supplementary information, Fig. S5b). The kinetics (Km) and Vmax for choline import by human Mfsd7c under this condition were 100 µM and 0.117 µmol/well/60 min, respectively. Choline uptake by the S203Y mutant in the absence of *hChKA* was completely inhibited (Fig. 2b, c). Interestingly, we noted that there was a slight increase in choline import in the S203Y mutant when co-expressed with *hChKA*, indicating that the transport activity of S203Y is severely delayed, but not abolished (Fig. 3d; Supplementary information, Fig. S5c). The Km and Vmax for importing choline by S203Y mutant under this condition were 1197 µM and 0.039 µmol/well/60 min, respectively. We also examined whether the expression of choline acetyltransferase (CHAT), a neuronal enzyme for acetylcholine synthesis, would increase choline uptake by converting choline into acetylcholine. Indeed, co-expression of CHAT and hMfsd7c greatly increased choline uptake (Fig. 3e). In the absence of ETNK1, an ethanolamine kinase, MFSD7c did not increase uptake of ethanolamine (Fig. 2k). However, transport of ethanolamine by MFSD7c was increased to a significant level when ETNK1 was co-expressed (Fig. 3f). Interestingly, co-expression of *hMfsd7c* with *hChKA* also slightly increased L-carnitine uptake (Fig. 3g). However, L-carnitine was unable to compete for choline import, suggesting that L-carnitine is a weak ligand (Supplementary information, Fig. S5d). Furthermore, we tested whether the transport of choline by MFSD7c is bidirectional. Indeed, our results showed that expression of hMfsd7c was required for the release of intracellular choline (Fig. 3h). Choline is a positively charged molecule. The import of choline would increase the membrane potential. To this end, we performed a whole-cell patch clamp of HEK293 cells co-expressing hMfsd7c or S203Y mutant with hChKA. Cells with overexpression of hChKA alone were used as a control (Supplementary information, Fig. S5e). Incubation of control cells or cells expressing S203Y mutant with 100 or 200 µM choline did not significantly increase membrane potential (Fig. 3i). In contrast, expression of *hMfsd7c* significantly increased membrane potential when 100 or 200 µM choline was included in the bath solution (Fig. 3i, j). Interestingly, the removal of choline reversed the increased membrane potential, suggesting that the influx of choline generates the membrane potential. These results show that the import of choline by MFSD7c is electrogenic. Collectively, our results show that MFSD7c facilitates the transport of choline and it behaves like a channel for the movement of choline via the plasma membrane.

### Mfsd7c mediates choline export at the BBB

**Fig. 4:**
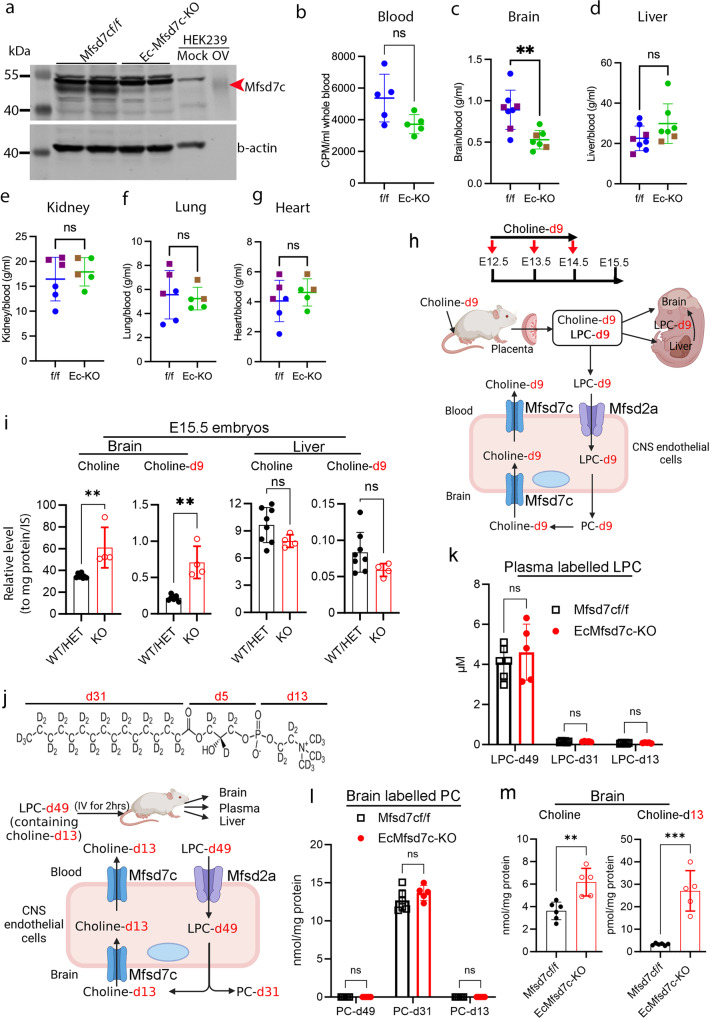
MFSD7c regulates choline levels in the brain. **a** Western blot analysis of the micro-vessels in the brains. Mfsd7c protein was reduced in Ec*Mfsd7c*-KO compared to controls (Mfsd7c^f/f^). Mock: 15 µg proteins from mock-transfected HEK239 cells; OV: 1.5 µg protein lysates from hMfsd7c overexpressed-HEK293 cells used for interpretation of Mfsd7c band. **b** Radioactive signals in the blood at 5 min post-injection of radioactive choline in Ec*Mfsd7c*-KO (Ec-KO) and control (f/f) mice. **c**–**g** The radioactive signals in the brain (**c**), livers (**d**), kidneys (**e**), lungs (**f**), and hearts (**g**) of Ec*Mfsd7c*-KO and control mice after 30 min of choline injection. There was a significant reduction of radioactive signals in the brain of Ec*Mfsd7c*-KO mice compared to controls. Each symbol represents one biological replicate. Circled and squared symbols indicate animals in the same batch of experiments. **P < 0.01; *t*-test. **h** Illustration of maternal deuterated choline delivery to the embryos. Choline-d9 is used for the synthesis of LPC-d9 by the maternal liver which is then delivered to the embryos. **i** Levels of endogenous choline and choline-d9 in the brains and livers of WT/heterozygous and KO embryos. Endogenous choline and choline-d9 were significantly increased in the brains but not in the liver of KO embryos. Each symbol represents one mouse. **P < 0.01, *t*-test. **j** Illustration of deuterated LPC-d49 tracing experiment in adult mice. Plasma LPC-d49 is taken up to the brain by Mfsd2a for PC synthesis. Choline-d13 released in the brains after the remodeling of LPC/PC-d49 is imported back to the endothelial cells by Mfsd7c. **k** Levels of LPC-d49, LPC-d31, and LPC-d13 in the plasma of controls and Ec*Mfsd7c*-KO mice at 2 h post-injection of LPC-d49. **l** Levels of PC-d49, PC-d31, and PC-d13 in the brains of controls and Ec*Mfsd7c*-KO mice at 2 h post-injection of LPC-d49. **m** Levels of endogenous choline and choline-d13 in the brains of controls and Ec*Mfsd7c*-KO mice at 2 h post-injection of LPC-d49. Each symbol represents one mouse. **P < 0.01, ***P < 0.001; *t*-test and two-way ANOVA (in **k** and **l**).

Choline uptake at the BBB has been documented in previous studies.^18–20^ However, the identity of the long-sought choline transporter was unknown. To test whether MFSD7c plays a role in choline transport in vivo, we generated conditional KO mice for *Mfsd7c* in the endothelial cells using *Cdh5*-*CreERT*^2^ (Fig. 4a). Next, we intravenously injected control (*Mfsd7c*^*f/f*^) and *Mfsd7c*^*f/f*^*Cdh5-CreERT*^2^ (hereafter, Ec*Mfsd7c*-KO) mice with a dose (2 mM in blood) of radioactive choline and measured the distribution of radioactive signals in brains, lungs, livers, hearts, and kidneys after 30 min. We chose this supraphysiological dose as the half-life of choline in plasma is within 3 min.^21^ The radioactive choline levels in blood were not different between the two genotypes after 5 min choline injection, indicating that choline was equally delivered to the blood of the mice (Fig. 4b). We found that radioactive signals were significantly reduced in the brains of Ec*Mfsd7c*-KO mice compared to control mice (Fig. 4c). However, we noted that the majority of injected choline was present in peripheral organs such as livers and kidneys; and the radioactivity in the organs was comparable between the genotypes, indicating that reduced choline import to the brain was specific in Ec*Mfsd7c*-KO mice (Fig. 4d–g). These results indicate that Mfsd7c can mediate the uptake of exogenous choline at the BBB, a result which is consistent with the findings in the literature.^18–20^ Nevertheless, the injected amount of choline was well above physiological concentrations of choline in blood.^22,23^ Perhaps, Mfsd7c mediates to import a small amount of choline to the brain at this supraphysiological dose. Of note, the radioactive signal in the brain accounted for 2%–3% of total radioactive signals.

Our metabolomic analysis showed that the levels of choline in the brain of *Mfsd7c*^–/–^ embryos were elevated (Fig. 1d). Mfsd7c is expressed in both sides of the CNS endothelial cells, which might suggest that Mfsd7c is also required for choline import from the brain parenchyma.^2^ To gain insights into the mechanism by which choline level is elevated in the brain of *Mfsd7c*-KO embryos, we injected deuterated choline (choline-d9) into the circulation of *Mfsd7c* pregnant dams and harvested *Mfsd7c*-KO embryos for lipidomic analysis of choline-containing lipids and choline (Fig. 4h). We found that the total levels of endogenous phospholipids such as LPC, phosphatidylcholine (PC), and sphingomyelin, as well as the deuterated phospholipids containing choline-d9 in the brains and livers of KO and control littermates, were comparable (Supplementary information, Table S11). Interestingly, we found that the levels of endogenous choline and choline-d9 were significantly elevated in the brains but not livers of KO embryos (Fig. 4i; Supplementary information, Table S11). Free choline imported to the brain is expected to be suppressed in the KO embryos. However, these results suggest that choline imported to the brains of *Mfsd7c*-KO embryos must be via other routes. We hypothesize that LPC delivers choline to the brain of *Mfsd7c*-KO embryos (Fig. 4h). The above data suggest a possibility that MFSD7c is also necessary for the export of choline from the brain parenchyma.

LPC is one of the most abundant lipids in the plasma^24,25^ and is imported to the brain by Mfsd2a via the BBB for phospholipid synthesis.^26,27^ To test our hypothesis that choline from LPC is accumulated in the brain of Ec*Mfsd7c*-KO mice, we injected a dose (300 µM in blood) of deuterated LPC-d49 which consists of choline-d13, palmitate-d31, and glycerol-d5 into the circulation of Ec*Mfsd7c*-KO and control mice. We then employed mass spectrometry (MS) for detection of choline-d13 and deuterated LPC and PC (Fig. 4j). In the plasma, a part of LPC-d49 was remodeled to produce LPC-d31 and LPC-d13. However, LPC-d49 was still the major deuterated LPC in the blood of the mice at 2 h post-injection (Fig. 4k). In the brain, we were able to detect deuterated PC species, which were present with similar amounts in the phospholipid pool of Ec*Mfsd7c*-KO and control mice. These results indicated that plasma LPC-d49 was equally imported to the brains for PC synthesis (Fig. 4l; Supplementary information, Table S12). Remarkably, we found that most of the deuterated PC species in the brains of the mice contained palmitate-d31 (PC-d31), instead of the intact form of LPC-d49 (PC-d49) (Fig. 4l; Supplementary information, Table S12). This indicated that there was a dramatic remodeling of plasma-derived LPC in the brain. As a result, the level of choline-d13 in the brains of Ec*Mfsd7c*-KO mice was significantly increased compared to that of control mice (Fig. 4m). Consistently, the levels of endogenous choline were confirmed to be elevated in Ec*Mfsd7c*-KO mice in the same analysis (Fig. 4m; Supplementary information, Fig. S6a, b), whereas the levels of choline-d13 in the plasma and livers of Ec*Mfsd7c*-KO and control mice were comparable, highlighting a specific elevation of choline in the brain of Ec*Mfsd7c*-KO mice (Supplementary information, Table S12). Furthermore, the levels of acetylcholine were increased in the brains of Ec*Mfsd7c*-KO adult mice, especially from mice fed with a choline-deficient diet (CDD) (Supplementary information, Fig. S6a, b). Since free choline import to the brain is likely suppressed or reduced in Ec*Mfsd7c*-KO mice (Fig. 4c), these results reveal an unexpected finding that excessive choline from LPC/PC metabolism is to be transported out of the brain and this mechanism is mediated by the activity of MFSD7c at the BBB.

### Missense mutations of *MFSD7c* result in reduced choline transport activity

**Fig. 5:**
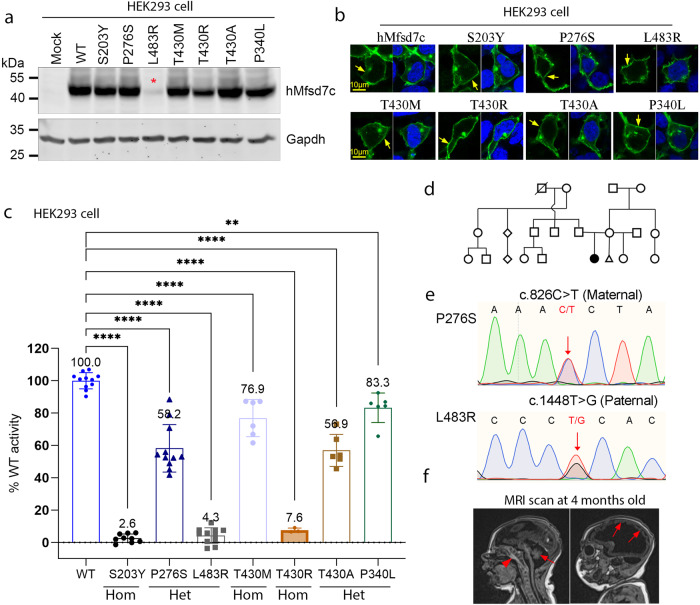
Mutations of *Mfsd7c* affect the choline transport function. **a** Western blot analysis of protein expression for the indicated missense mutations. **b** Immunofluorescent analysis of the localization of the indicated mutant proteins in HEK293 cells. **c** Missense mutations of *Mfsd7c* affect choline import activity. Percentage of choline transport activity of the indicated mutants compared to WT Mfsd7c. Data are expressed as mean ± SD. Each symbol represents one replicate.****P < 0.0001, **P < 0.01; one-way ANOVA. Mean percentage of transport activity for each mutant was shown on top of each bar. **d** Pedigree of the patient’s families. **e** Sanger sequencing results confirm the mutation P276S from the mother and L483R from the father in a non-consanguinity family. **f** MRI images of the patient taken at 4 months of age. The arrowhead shows signs of calcification. The arrows show delayed development of the cerebellum and neocortex.

Missense mutations of *MFSD7c* have been reported in patients with Fowler syndrome. However, it remains unknown whether these mutations cause a loss or gain of choline function of *MFSD7c*. Thus, we performed mutagenesis to generate several homozygous and compound heterozygous missense mutations including S203Y, T430R, and T430M, and tested their transport activity (Fig. 5). These homozygous missense mutations S203Y, T430R, and T430M were chosen because the first two former mutations caused lethal Fowler syndrome, whereas the latter mutation caused non-lethal Fowler syndrome.^1,2,28,29^ We also examined the activity of missense mutations P340L and T430A that were reported in a non-lethal Fowler patient.^2^ First, we confirmed that these missense mutations did not affect the expression and localization of MFSD7c, except for L483R (Fig. 5a, b). Second, we measured choline transport activity and found that most of these missense mutations of *MFSD7c* exhibited significantly reduced or abolished choline transport activity (Fig. 5c). A patient homozygous for S203Y was aborted at 20 weeks during gestation.^2^ Choline transport activity of this missense mutation was abolished (Fig. 5c). The T430M mutation was associated with non-lethal Fowler syndrome, whereas homozygous T430R mutation was reported to be lethal.^1,4^ We showed that T430M had ∼76.9% activity compared to that of WT protein, whereas T430R is an inactive mutant (Fig. 5c). Previously, compound heterozygous mutants (P340L and T430A) were found in a 2-year-old patient, who is still living.^2^ His brain Magnetic Resonance Imaging (MRI) showed mild microcephaly. We found that both alleles had partially decreased choline transport activity (Fig. 5c). The current study also identified a patient with phenotypes associated with Fowler syndrome. Whole exome sequencing identified two missense mutations of the *MFSD7c* coding sequence that led to a substitution of proline at position 276 to serine (P276S) inherited from the mother and leucine at position 483 to arginine (L483R) from the father (Fig. 5d–f). The patient exhibited severe microcephaly with neurological disorders (Fig. 5f). Transport activity of these missense mutants showed that P276S retained 58.2% choline transport activity, whereas L483R is likely unstable, leading to a severe reduction of activity in MFSD7c (Fig. 5c). Expending these results, we also tested the missense mutations that have been reported in the literature. We found that most lethal mutations caused a significant reduction of choline transport activity (Supplementary information, Fig. S7 and Table S13). Nevertheless, several missense mutations led to a partial loss of choline transport activity, but the patients carrying these mutations exhibited severe clinical features. It is unclear whether other factors also contribute to the pathological conditions in these cases. Together, these results suggest that reduced choline transport activity of MFSD7c may be associated with the pathogenesis of Fowler syndrome. The discovery of the molecular function of MFSD7c as a choline transporter is an important step to facilitate the understanding of the disease mechanisms in patients with Fowler syndrome.

Although our results show that accumulation of choline might be a causal factor, we took a systemic approach to address whether deficiency of choline or choline accumulation in the brain is linked to the phenotypes. First, if choline is imported into the brain for phospholipid synthesis, it is expected that the brain phospholipid profile of *Mfsd7c* KO mice must be reduced. We performed a lipidomic analysis of the brains from *Mfsd7c* KOs and controls. However, there were no significant changes in the levels of major phospholipids containing choline such as LPC, PC, and SM in *Mfsd7c* KO embryos and adult mice (Supplementary information, Fig. S8a, b and Tables S14–S17). Additionally, we compared the brain transcriptomes of KO embryos to those of WT embryos obtained from dietary choline-deficient mice as we argued that deficiency of choline would recapitulate the deletion of *Mfsd7c* (Supplementary information, Fig. S9 and Table S18). Nevertheless, we did not observe any significant change in gene expression from WT embryos obtained from dams fed with a CDD compared to that of *Mfsd7c* KO embryos. These results indicate that deficiency of choline is unlikely to be the cause for the phenotypes in the brain of *Mfsd7c*^–/–^ embryos. Second, we tested whether the depletion of choline from diets would improve the phenotypes in *Mfsd7c*^–/–^ embryos. We depleted choline from the diets of pregnant *Mfsd7c*^–/–^ mice and examined the CNS blood vessels of *Mfsd7c*^–/–^ embryos. We found that the depletion of choline slightly, but significantly reduced the dilation of blood vessels in the cortices of E14.5 *Mfsd7c*^–/–^ embryos (Supplementary information, Fig. S10). Nonetheless, maternal choline deficiency did not prevent the migratory defects of the blood vessels to the ganglionic eminence regions of these *Mfsd7c*^–/–^ embryos (Supplementary information, Fig. S10d), probably due to the dominant source of choline from LPC. Thus, a dietary intervention approach to reduce brain choline levels might not be an effective approach to reverse the phenotypes of *Mfsd7c*^–/–^ embryos.

## Discussion

The import of choline to the brain via the BBB was evidenced several decades ago.^18–20,30^ Most of these prior studies used in situ rat models and reported that exogenous choline is rapidly taken up to the brain. These experiments were often stopped within 1–3 min and the whole brain was collected without perfusion.^18,19,30^ Although these studies inferred that plasma choline was imported to the brain via the BBB, it was rather elusive whether choline is taken up to the brain at physiological conditions. To our knowledge, the molecular machinery for choline import at the BBB as well as the source of choline anticipated by these prior studies remains uncharacterized. MFSD7c is specifically expressed in endothelial cells of the CNS blood vessels.^20,31–33^ Deletion of *Mfsd7c* in endothelial cells recapitulates the phenotypes obtained from the global KO, implicating its major role at the BBB.^7^ The current work identifies MFSD7c as a choline transporter, which facilitates choline transport bidirectionally. Employing *Mfsd7c* KO models, we observe that injection of a high dose (2 mM) of radioactive choline to the blood increases choline import to the brain via MFSD7c. This result recapitulates the prior reports in the literature and suggests that if a high level of choline is delivered to the blood, a small amount of choline might be imported to the brain by MFSD7c. Nevertheless, the import function of choline by MFSD7c might not be relevant in physiological conditions because a higher level of choline present in the blood might force the transport direction toward the brain side. In our in vivo transport experiments, we detected approximately 2%–3% injected choline in the brain amongst these analyzed organs (not all organs were collected). These results are well in line with numerous reports using radioactive choline (11C or 18F-choline) for cancer diagnosis. Most of radioactive choline was accumulated in the peripheral organs such as liver, kidney, lung, and prostate.^34–36^ Consistently, the amount of injected choline present in the brain accounts for approximately 1%–3% of total injected choline in humans.^34^ Thus, it seems that the brain is not equipped with an effective machinery to actively import blood choline in comparison with peripheral tissues. Furthermore, the half-life of choline in the circulation is 1–3 min. It is believed that the liver sequesters blood choline for the biosynthesis of PC and LPC in which the latter is then released into the circulation. Thus, it might be that the detected amount of radiolabelled choline imported to the brain of mice and humans contains a portion of LPC and/or other choline metabolites such as glycerolphosphorylcholines (GPC). These findings argue against the previously established notion that blood choline is imported into the brain.

Employing *Mfsd7c* KO mice, we unexpectedly discover that the expression of MFSD7c at the CNS blood vessels is required for the export of choline from the brain. We demonstrate that plasma LPC is a carrier of choline to the brain. Within the brain, the remodeling of phospholipids derived from LPC liberates choline. Our data show that LPC-derived choline is accumulated in the brain of *Mfsd7c* KO mice, implicating that an excessive amount of choline is exported out of the brain. The levels of acetylcholine were slightly increased in *Mfsd7c* KO mice, suggesting that the increased choline is used for acetylcholine synthesis. We argue that LPC-Mfsd2a pathway is an active pathway that likely provides a sufficient amount of choline in the brain. The levels of plasma LPC can reach 400–500 µM,^25^ whereas plasma choline level is ∼10–15 µM.^22,23^ Plasma LPC is transported to the brain by Mfsd2a which is one of the most abundant genes expressed in the BBB.^26,32,37,38^ These data point to the conclusion that LPC provides a major source of choline to the brain, not free choline from the plasma. Thus, it is likely that the major physiological function of MFSD7c at the BBB is to reduce excess choline in the brain.

At this point, it is still unclear whether MFSD7c exports brain choline to the plasma or choline is accumulated in the endothelial cells. The increased levels of acetylcholine in the brain of *Mfsd7c* KO mice might hint that at least a portion of choline is indeed accumulated in the brain parenchyma since acetylcholine is mainly generated by cholinergic neurons.^39^ MFSD7c is also expressed at the abluminal side of the endothelial cells. Thus, the excessive amount of choline may be delivered to the endothelial cells by MFSD7c. Based on our results, it is suggested that the accumulation of choline in the brain due to *Mfsd7c* mutations might be a causal factor for the phenotypes. The dietary choline depletion approach has been applied to test whether the accumulation of choline would be a detrimental factor. However, this approach could not eliminate the contributions of brain choline from other pathways such as LPC-Mfsd2a or GPC, resulting in a modest improvement of the phenotypes. Thus, the pathological consequences of choline accumulation to the defective BBB of *Mfsd7c* KO mice remain to be investigated.

In summary, our work identifies Mfsd7c as a choline transporter at the BBB. This study provides a foundation for future investigation on a potential mechanism linking the defective choline export function of MFSD7c to the pathogenesis of Fowler syndrome. The discovery of MFSD7c as a choline exporter at the BBB reveals an unexpected route of choline transport between blood and brain and opens up new avenues delving into the metabolic fates of choline in the brain.

## Materials and methods

### Mice

Global deletion of *Mfsd7c* has been described previously.^2^ To generate the postnatal deletion of *Mfsd7c* in the BBB, *Mfsd7c*^f/f^ was crossed with *Cdh5-Cre*^*ERT*2^ mice^40^ to generate *Mfsd7c*^f/f^*Cdh5-Cre*^*ERT2*^ mice (Ec*Mfsd7c*-KO). At about 2 months of age, mice were injected daily with 5 doses of 200 µg/g bodyweight tamoxifen prepared in corn oil via oral gavage.^41^ Male and female mice with age more than 8 weeks old were used for experiments. Mice were maintained at a constant temperature of 20 °C with 12-h light/12-h dark cycle on normal chow diets. All experimental protocols and procedures in protocol R19-0567 and R23-0973 were approved by IACUC committees under National University of Singapore.

### Plasmids

All plasmids were constructed in pcDNA3.1 vector for overexpression unless stated otherwise. *hChKA* (D10704.1), *ETNK1*, *CHAT* (NM_020986.4) genes were synthesized from GenScript and inserted into pcDNA3.1, respectively. *pcDNA3.1-hMfsd7c*, *pcDNA3.1-hMfsd7cT430M* and *S203Y* were generated previously.^2^ The other missense mutations including hMfsd7cT430A, P276S, L483R, P340L, and T430R were generated by mutagenesis and confirmed by Sanger sequencing. Zebrafish (*DaMfsd7c_a*, XM_688497.7; *DaMfsd7c_b*: XM_003200755.5), medaka fish (*MeMfsd7c*, XM_004082328.4), and frog Mfsd7c (*XeMfsd7c*, NM_001016982.2) coding sequences were synthesized by GenScript and cloned into pcDNA3.1 plasmid for overexpression, respectively. The *pESC-HIS-hMfsd7c* plasmid (GenScript) was used for overexpression in *S. cerevisiae*.

### Untargeted metabolite analysis

Untargeted metabolomics analysis was performed at the Genome Analysis Center, Research Unit Molecular Endocrinology and Metabolism, Helmholtz Center Munich. Frozen WT and *Mfsd7c* KO embryonic brain and liver samples at E14.5 were weighed and placed into pre-cooled (dry ice) 2 mL homogenization tubes containing ceramic beads with a diameter of 1.4 mm (Precellys® Keramik-Kit 1.4 mm). Pre-cooled water with a ratio of 5 µL/mg tissue was added into the tubes. The samples were then homogenized in Precellys 24 homogenizer (PEQLAB Biotechnology GmbH, Germany) equipped with an integrated cooling unit 3 times for 20 s at 5500  rpm each, with 30 s intervals (to ensure freezing temperatures in sample vials) between the homogenization steps. 100 µL of the brain and liver homogenates were loaded onto two separate 2 mL 96-deep well plates, one plate for the brain sample set and the other for the liver samples. Three types of quality control samples were analyzed in concert with the experimental samples in each sample set: samples generated from a pool of human plasma; samples generated from a small portion of each experimental sample served as technical replicates throughout the dataset; and extracted water samples served as process blanks. Experimental samples and controls were randomized across the metabolomics analysis.

The homogenates in each well of the 2 mL 96-deep well plate were extracted with 500 µL methanol, containing four recovery standards to monitor the extraction efficiency. After centrifugation, the supernatant was split into aliquots in two 96-well microplates. The first 2 aliquots of each sample set were used for ultra-high performance liquid chromatography-tandem mass spectrometry (UPLC-MS/MS) analysis in positive electrospray ionization modes (i.e., early and late eluting compounds). 1 aliquot of each sample set was used for UPLC-MS/MS analysis in negative ionization mode, and the rest were kept as reserve for backup. The extract aliquots were dried on a TurboVap 96 (Zymark).

Prior to UPLC-MS/MS analysis, the dried extract samples were reconstituted in acidic or basic LC-compatible solvents, each of which contained eight or more standard compounds at fixed concentrations to ensure injection and chromatographic consistency. The UPLC-MS/MS platform utilized a Waters Acquity UPLC with Waters UPLC BEH C18-2.1 × 100 mm, 1.7 μm columns and Q-Exactive high resolution/accurate mass spectrometer (ThermoFisher Scientific) interfaced with a heated electrospray ionization (HESI-II) source and Orbitrap mass analyzer operated at 35,000 mass resolution. Extracts reconstituted in acidic conditions were gradient eluted using water and methanol containing 0.1% formic acid, while the basic extracts, which also used water/methanol, contained 6.5 mM ammonium bicarbonate. The MS analysis alternated between MS and data-dependent MS2 scans using dynamic exclusion, and the scan range was from 80 to 1000 m/z.

Metabolites were identified by automated comparison of the ion features in the experimental samples to a reference library of chemical standard entries that included retention time, molecular weight (m/z), preferred adducts, and in-source fragments as well as associated MS spectra and curated by visual inspection for quality control using software developed at Metabolon. Chromatographic peaks were quantified using area-under-the-curve.

### In vivo transport of radiolabelled choline

To study the uptake of choline into the brain, control (*Mfsd7c*^*f/f*^) and Ec*Mfsd7c*-KO mice aged 4–6 months were intravenously injected with 2 mM radiolabelled choline (stock: 333 mM choline with 0.05 µCi/µL). After 5 min of injection, 50 µL blood was collected and the radioactive levels were quantified to monitor the initial radiolabelled levels in the circulation. After 30 min of injection, mice were perfused with 20 mL of PBS. Then, brains, lungs, hearts, kidneys, and livers were collected for quantification of radioactive signals. A similar amount of tissues was homogenized in RIPA buffer and the radioactive signals were quantified by a liquid scintillation counter. The radioactive signals from tissue from each mouse were normalized to radioactive signals from blood collected at 5 min post-injection.

### Choline-d9 and LPC-d49 experiments

For stable isotopic tracing with choline-d9 (containing 9 deuterium atoms) injection, pregnant *Mfsd7c*^*+/–*^ mice at 12.5 days of gestation were injected daily by intravenous route with an amount of 2 mM choline-d9/day until gestation day 14.5 (total of 3 doses of choline-d9 from E12.5–14.5). The embryos were collected at E15.5 after the last dose at E14.5 and genotyped. The brains and livers of embryos were harvested for lipid extraction for lipidomic analysis. Briefly, an equal amount of brain and liver lysates from WT, heterozygous and KO embryos were extracted with 1-butanol:methanol (ratio 1:1) method for choline and lipid analyses by LC-MS/MS.

For stable isotopic LPC-d49 (870308P-5MG, Sigma-Aldrich, containing choline-d13, glycerol-d5, and palmitate-d31) injection, Ec*Mfsd7c*-KO and control mice aged 7–10 months were injected with a dose of 300 µM LPC-d49 in 12% BSA. After 2 h of injection, plasma was collected from the blood; then the mice were perfused with cold PBS to remove blood. After that the brains and livers were collected. Brains and livers were homogenized in PBS and the same amount of tissue lysates were used for lipid and choline extraction with chloroform:methanol (1:2 v/v) method.^42^ Organic phase was used for lipid analysis and the aqueous phase (upper phase) was used for choline analysis by LC-MS/MS.

### Lipids and choline quantification by LC-MS/MS

Samples were randomized before extraction and analysis. Blank samples (10 µL MilliQ water), matrix blanks (samples extracted without spiking internal standards, see below), and pooled QC samples were used to assess method performance. In addition, diluted pooled QC samples were used to assess response linearity. Blanks, blank extracts, QC, and diluted QC were interspersed with study samples throughout the analytical run. Phospholipids and choline were separated using HILIC column (Kinetex 2.6 µm HILIC 100 Å, 150 × 2.1 mm, Phenomenex, Torrance, CA, USA) on Agilent 6495A and 6495C triple quadrupole mass spectrometers (Agilent Technologies). Mobile phases A (50% acetonitrile (LC-MS grade, ThermoFisher Scientific) and 50% 25 mM ammonium formate (Sigma-Aldrich), pH 3.5) and B (95% acetonitrile and 5% of 25 mM ammonium formate, pH 3.5) were mixed at the following gradient: 0–6 min, 99%–75% B; 6–7 min, 75%–10% B; 7–7.1 min, 10%–99% B; 7.1–10.1 min, 99% B. The flow rate was 0.6 mL/min, and the sample injection volume was 1 µL. MS parameters were as follows: electrospray ionization, gas temperature 200 °C, gas flow 12 L/min, sheath gas flow 12 L/min, and capillary voltage 3500 V. Phospholipids were quantified at the sum composition level using multiple reaction monitoring (MRM) using transition to phosphocholine headgroup (*m/z* 184 for endogenous lipids and adjusted to appropriate *m/z* for deuterated lipids). Choline was quantified using MRM using a transition from 104 to 60 (adjusted to appropriate *m/z* for deuterated choline). These internal standards (ISs) were used for lipidomics and choline analysis by LC-MS/MS. IS solutions were prepared in butanol/methanol (BuMe) (1:1, v/v) or chloroform/methanol (1:2) containing phospholipid IS SPLASH Mix (Avanti, 330707) (containing 5.3 µM 15:0–18:1(d7) PC, 0.19 µM 15:0–18:1(d7) PE, 0.13 µM 15:0–18:1(d7) PS (Na Salt), 0.93 µM 15:0–18:1(d7) PG (Na Salt), 0.27 µM 15:0–18:1(d7) PI (NH_4_ Salt), 0.27 µM 15:0–18:1(d7) PA (Na Salt), 1.20 µM 18:1(d7) Lyso PC, 0.27 µM 18:1(d7) Lyso PE, 13.32 µM 18:1(d7) d18:1–18:1(d9) SM). Choline-d9 was used as IS in choline analysis.

### Transport assays in HEK293 cells

*hMfsd7c* or mouse *mMfsd7c* cDNA constructed in pcDNA3.1 was transfected to HEK293 cells using lipofectamine 2000 (ThermoFisher Scientific). Mock was treated with an empty plasmid. For experiments in which *hChKA* was used, 1–1.5 µg *pcDNA3.1-hChKA* plasmid was co-transfected with 2–2.5 µg plasmid of *hMfsd7c* or mutant plasmids. Cells transfected with 1–1.5 µg *pcDNA3.1-hChKA* plasmid were used as control. After 18–30 h post-transfection, cells were used for transport assays with 100 µM [^3^H] choline (ARC: ART 0197) in DMEM containing 10% FBS. The transport assays were stopped after 1 h incubation at 37 °C by washing once with cold plain DMEM medium. Cell pellets were lyzed in RIPA buffer and transferred to scintillation vials for quantification of the radioactive signal using Tricarb liquid scintillation counter. The transport activity of MFSD7c was expressed as DMP for [^3^H] isotopes and CPM [^14^C] isotopes. For dose-dependent transport activity, HEK293 cells were similarly transfected with *hMfsd7c* or *hMfsd7cS203Y* mutant plasmid alone or co-transfected with *hChKA*. Transport activity was conducted with 20, 45, 90, and 250 µM [^3^H] choline in DMEM containing 10% FBS for 1 h at 37 °C. For the time-dependent assay, *hMfsd7c* was transfected or co-transfected with *hChKA*. Transfected HEK293 cells were incubated with 100 µM [^3^H] choline in DMEM containing 10% FBS for 15, 30, 60, and 120 min at 37 °C. For transport assay with [^14^C] ethanolamine, [^3^H] L-carnitine, and [^3^H] acetyl-carnitine, [^3^H] palmitoyl-carnitine, [^14^C] octanoyl-carnitine, HEK293 cells were co-transfected with *MFSD7c* and *hChKA* as described above. Transport assays were performed with 100 µM [^14^C] ethanolamine, 1 mM [^3^H] L-carnitine, 1 mM [^3^H] acetyl-carnitine, 10 µM [^3^H] palmitoyl-carnitine, or 100 µM [^14^C] octanoyl-carnitine in DMEM with 10% FBS for 1 h. Cells were washed once with cold plain DMEM and lyzed with RIPA buffer for radioactive quantification. For import assays with the missense mutants, these plasmids were co-transfected with *hChKA* to HEK293 cells. Import assays were performed with 100 µM [^3^H] choline in DMEM containing 10% FBS for 60 min. Radioactive signals from cells were quantified and converted to a percentage of WT activity.

For export assays, HEK293 cells were transfected with empty, *hMfsd7c*, or *mMfsd7c* plasmids. After 24 h of transfection, cells were loaded with 500 µM [^3^H] choline for 1 h. The cells were then washed to remove the remaining [^3^H] choline in the medium and incubated with plain DMEM medium to stimulate the release. The cells were harvested at 0 (before release) and 1 h (after release) after incubation with DMEM for radioactive quantification as described above.

### Sodium and pH-dependent transport assays

For sodium-dependent assays, sodium is replaced with lithium in transport buffer (NaCl buffer: 150 mM NaCl, 5 mM KCl, 10 mM HEPES-Na, pH 7.4; LiCl buffer: 150 mM LiCl, 5 mM KCl, 10 mM HEPES, pH 7.4 with HCl). For pH-dependent assays, NaCl buffer was adjusted to pH 6.5 and pH 8.5, respectively. For transport assay conditions, overnight *hMfsd7c* and *hChKA* co-transfected HEK293 cells were washed once with the same buffer before being incubated with 100 µM [^3^H] choline for 15 min at 37 °C. Cells were lyzed in RIPA buffer and mixed with scintillation fluid for radioactive quantification.

### Electrophysiology

For single-cell recording of membrane potential alteration during transport of choline, HEK293FT cells were cultured in DMEM medium supplemented with 10% FBS and 1% penicillin and streptomycin and maintained in 5% CO_2_ incubator at 37 °C. For transfection, cells were seeded on the Petri dishes and grown overnight. Subsequently, 2 µg of empty *pIRES2-EGFP* (empty plasmid), *pIRES2-EGFP-hMfsd7c* or mutant *pIRES2-EGFP-S203Y* plasmid was co-transfected with 2 µg of *pIRES2-RFP-hChKA* plasmid using lipofectamine 2000. The transfected cells were incubated for 24 h in 5% CO_2_ incubator at 37 °C. At 48 h post-transfection, the cells were split and seeded on poly-_D_-lysine-coated coverslips for one more day before recording.

### Whole-cell patch clamp recordings and data analysis

To record the changes in resting membrane potential, the internal solution (pipette solution) contained 130 mM K-gluconate, 10 nM KCl, 5 mM EGTA, 10 mM HEPES, 1 mM MgCl_2_, 0.5 mM Na_3_GTP, 4 mM Mg-ATP, 10 mM Na-phoshocreatine, pH 7.4 (adjusted with KOH) was filled in the pipette tip. The external solution contained: 10 mM Glucose, 125 mM NaCl, 25 mM NaHCO_3_, 1.25 mM NaH_2_PO_4_·2H_2_O, 2.5 mM KCl, 1.8 mM CaCl_2_, 1 mM MgCl_2_, pH 7.4 (300–310 mOsm). Single cells with both EGFP (empty, *hMfsd7c* or *S203Y* plasmid) and RFP (*hChKA*) fluorescence were visualized under a fluorescent microscope for patching. Resting membrane potential was recorded under the current clamp with an Axopatch200B or multiclamp 200B amplifier (Molecular Device) with 0 pA current injection. Subsequently, 200 µM choline was perfused to the cells after 2 min of stable baseline recording. After 5 min of recording, the ligand was washed away. Approximately, 10 individual cells were recorded for each condition. The data were analyzed using GraphPad Prism V software (San Diego, CA) and Microsoft (Seattle, WA) Excel. Data are expressed as mean ± SEM.

### Expression of *hMfsd7c* in *S. cerevisiae Hnm1* mutants

*S. cerevisiae* acquires choline via HNM1. The *S. cerevisiae Hnm1* mutants were acquired from Dharmacons. WT and *Hnm1* mutant yeasts were grown in Yeast extract-Peptone-Dextrose (YPD) medium at 30 °C overnight before transformation. The *pESC-HIS-hMfsd7c* plasmid was transformed into WT and *Hnm1* mutant yeasts following the previously described protocol.^43^ After heat shock at 42 °C for 30 min and recovery in YPD for 2 h, the yeasts were spread on 2% agar plates prepared in yeast nitrogen base (YNB) medium (Sigma-Aldrich) supplemented with 2% glucose and yeast synthetic drop-out medium supplements without histidine (Sigma-Aldrich). Positive colonies were picked up randomly and further confirmed by transport assay and western blot.

### Transport assay for yeast cells

WT, *Hnm1* mutant and transformed yeast cells were grown in YNB medium with 2% glucose and drop-out medium supplements without histidine at 30 °C overnight followed by induction of hMfsd7c expression in YNB medium with 2% galactose for at least 5 h at 30 °C. An 800 µL of yeast cells at OD_600_ = 1 was centrifuged, washed twice with distilled water, then incubated in PBS containing 100 µM [^3^H] choline for 10 min. The transport assay was stopped by washing twice with PBS containing 100 µM choline. Yeast cells were lyzed with lysis buffer containing 2% Triton-X, 1% SDS, 100 mM NaCl, 10 mM Tris-HCl (pH 8.0), 1 mM EDTA (pH 8.0) at 95 °C for 5 min for radioactive quantification by liquid scintillation counter.

### Western blot analysis

For protein extraction, transfected HEK293 cells with *hMfsd7c*, *mMfsd7c*, *hMfsd7c* mutants were lysed with RIPA buffer, respectively. For validation of deletion of *Mfsd7c* in mice, micro-vessels from PBS-perfused brains of WT and Ec*Mfsd7c*-KO mice were isolated, homogenized and lyzed with RIPA buffer. For Western blot analysis of hMfsd7c expression in yeasts, 1 mL of yeast culture at OD_600_ = 1 from WT, *Hnm1* mutant yeasts or transformed yeasts was used for total protein extraction. BCA assay was used for total protein quantification. These antibodies were used: VDAC (Cell Signaling, CS4866), CoxIV (Invitrogen, MA5-15686), OPA1 (BD biosciences, 612602), MRPS35 (Protein biotech, 16457), CHKA (Cell Signaling, CS13422S), HMOX1 (Proteintech, 10701-1-AP), NDUFS1 (Proteintech, 12444-1-AP). Western blot analysis was conducted as previously described.^2^

### Fowler patient recruitment

A 5-month-old infant born at 39w1d with multifocal epilepsy, ventriculomegaly, developmental delay, and lissencephaly was identified via GeneMatcher collaboration.^44^ She was born to a 38-year-old mother. Pregnancy was complicated by gestational diabetes and maternal hypertension. There was concern for lissencephaly on prenatal ultrasound and MRI. Prenatal CMA and karyotype were normal. She was delivered vaginally complicated by shoulder dystocia. She required brief positive pressure ventilation and was admitted to the NICU on NIPPV. Apgars at 1 and 5 min were 4 and 9, respectively. Birth parameters include length of 49 cm (47th percentile WHO), weight of 2.86 kg (20th percentile WHO), and head circumference of 32 cm (6th percentile WHO). Her whole exome sequencing at birth showed two variants of uncertain significance in *FLVCR2*, c.826C>T (Maternal) and c.1448T>G (Paternal). The missense mutations were confirmed by Sanger sequencing. There was no family history of a similar presentation. This was the first pregnancy between this patient’s parents. Mother had a previous miscarriage and a healthy older daughter. Parents report no consanguinity. The IRB approval for this case study was exempted by the Human Research Protection Office (HRPO) of Washington University in St. Louis.

### RNA sequencing and analysis

For bulk RNA sequencing of whole brains from E14.5 WT and KO embryos from normal chow diet, this dataset has been deposited in GEO database with the accession number: GSE148854. For RNA sequencing of isolated endothelial cells from heterozygous and KO embryos at E14.5, we re-analyzed the dataset GSE129838.^7^ For RNA sequencing of whole brains from E14.5 WT embryos from CDD, pregnant WT mice were fed with CDD at the mating day until embryo collection. The whole brain of E14.5 embryos was collected for bulk RNA sequencing. This dataset has been deposited in the GEO database with the following accession number: GSE239589.

### Immunofluorescence staining for neocortical vessels

*Mfsd7c*^*+/–*^ female mice were fed on CDD for 2 weeks and then time-mated with the heterozygous male. E14.5 embryos were dissected from the pregnant female for collection of brains for immunostaining of CNS vessel morphology. Brain section was permeabilized in 0.5% triton X-100 (PBST), blocked in 5% normal goat serum and then incubated with anti-mouse GLUT1 (Abcam, ab40084,1:200) at 4 °C overnight, followed by 3 washes in PBS at 10-min intervals. Then, the goat anti-mouse antibody (Alexa Fluor 488, ThermoFisher Scientific, A11034,1:500) was added to the slides for 1-h incubation at room temperature to visualize the GLUT1 signal. Images were taken by the confocal microscope Zeiss LSM710. Vessel phenotypes were analyzed using measurement tools in ImageJ.

### Choline and acetylcholine assays

Choline and acetylcholine from plasma, liver, and brain samples were measured by fluorometric assay (Abcam: ab65345). Tissues were homogenized in choline buffer. Choline and acetylcholine were assayed according to the manufacturer’s instructions.

### Transmission electron microscope (TEM)

WT (*n* = 3) and *Mfsd7c*^–/–^ (*n* = 3) embryos at E14.5 were collected from the same pregnant mice. Samples were fixed with a solution containing 2.5% glutaraldehyde in 0.1 M PBS buffer (pH 7.3). Samples were then washed and post-fixed with 1% buffered osmium. The samples were dehydrated in increasing concentrations of ethanol and then infiltrated and embedded in Araldite medium. The samples were then polymerized in a 60 °C oven for approximately 2 days. Ultrathin sections were cut using a Leica Ultracut microtome and then stained with lead citrate. The stained samples were examined in a JEM 1400 transmission electron microscope (JEOL USA, Inc., Peabody, MA) using an accelerating voltage of 100 kV. Digital images were obtained using a CMOS Matataki Flash 2K CCD camera.

### Statistical analysis

Data were analyzed using GraphPrism9 software. Statistical significance was calculated using parametric and non-parametric *t*-tests, one- or two-way ANOVA as indicated in the figure legends. *P* < 0.05 was considered as statistically significant.

### Supplementary information

Supplementary information Fig S1

Supplementary information Fig S2

Supplementary information Fig S3

Supplementary information Fig S4

Supplementary information Fig S5

Supplementary information Fig S6

Supplementary information Fig S7

Supplementary information Fig S8

Supplementary information Fig S9

Supplementary information Fig S10

Supplementary Tables 1–5

Supplementary Tables 6–10

Supplementary Table 11

Supplementary Table 12

Supplementary Table 13

Supplementary Table 14

Supplementary Table 15

Supplementary Table 16

Supplementary Table 17

Supplementary Table 18

## Data Availability

All data have been included in the manuscript.
