# Supplementary material for: MFSD7c functions as a transporter of choline at the blood–brain barrier
Source: Cell Res. 2024 Feb 2;34(3):245–57. doi: 10.1038/s41422-023-00923-y (PMC10907603; doi:10.1038/s41422-023-00923-y)
Supplement: Supplementary file 1 — Supplementary information Fig S1 [file 41422_2023_923_MOESM1_ESM.pdf]

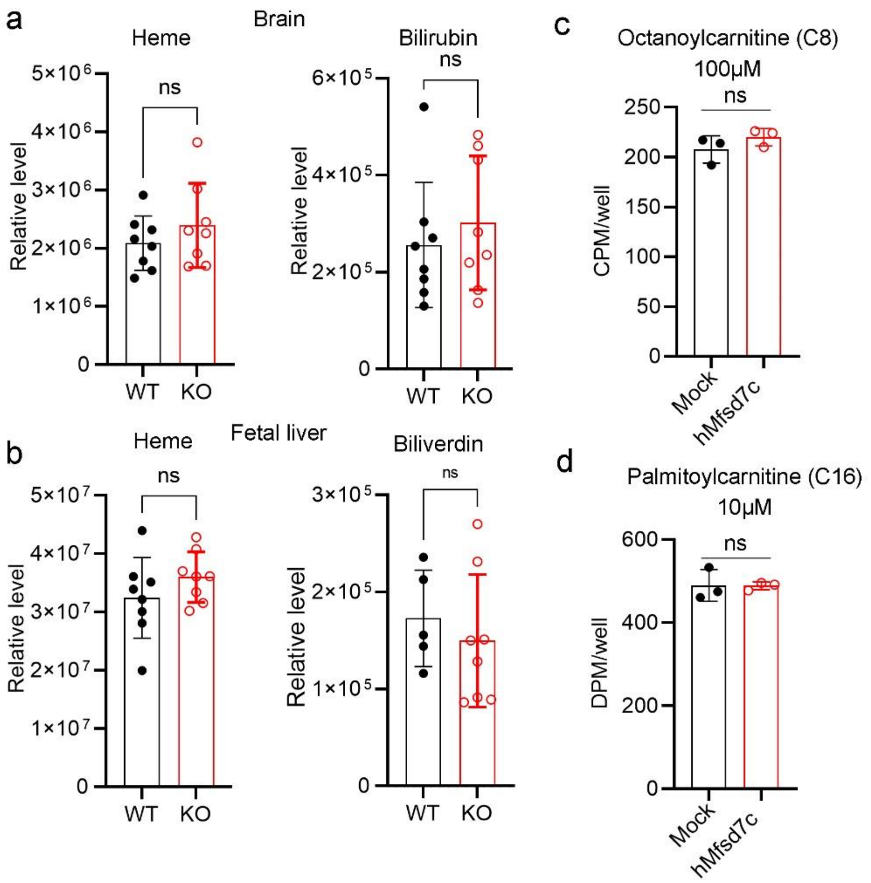

Supplementary information, Fig. S1. Comprehensive metabolite analysis did not detect changes in heme and heme metabolites in the brain of Mfsd7c knockout, related to Figure 1. ***a,*** *heme and bilirubin levels in the brains of WT and Mfsd7c$^{-/-}$ (KO) embryos.* ***b****, heme and biliverdin levels in the livers of WT and Mfsd7c$^{-/-}$ (KO) embryos. Each symbol represents on embryo. ns, not significant.* ***c-d****, Import assays for octanoylcarnitine and palmitoylcarnitine in HEK239 cells overexpressed with hMfsd7c. Mfsd7c did not import these long chain acyl-carnitines. Experiments were repeated thrice in triplicate. ns, not significant. t-test was used. Full list of metabolites can be found in the* ***Supplementary information, Tables S1-10****.*
