# Supplementary material for: MFSD7c functions as a transporter of choline at the blood–brain barrier
Source: Cell Res. 2024 Feb 2;34(3):245–57. doi: 10.1038/s41422-023-00923-y (PMC10907603; doi:10.1038/s41422-023-00923-y)
Supplement: Supplementary file 2 — Supplementary information Fig S2 [file 41422_2023_923_MOESM2_ESM.pdf]

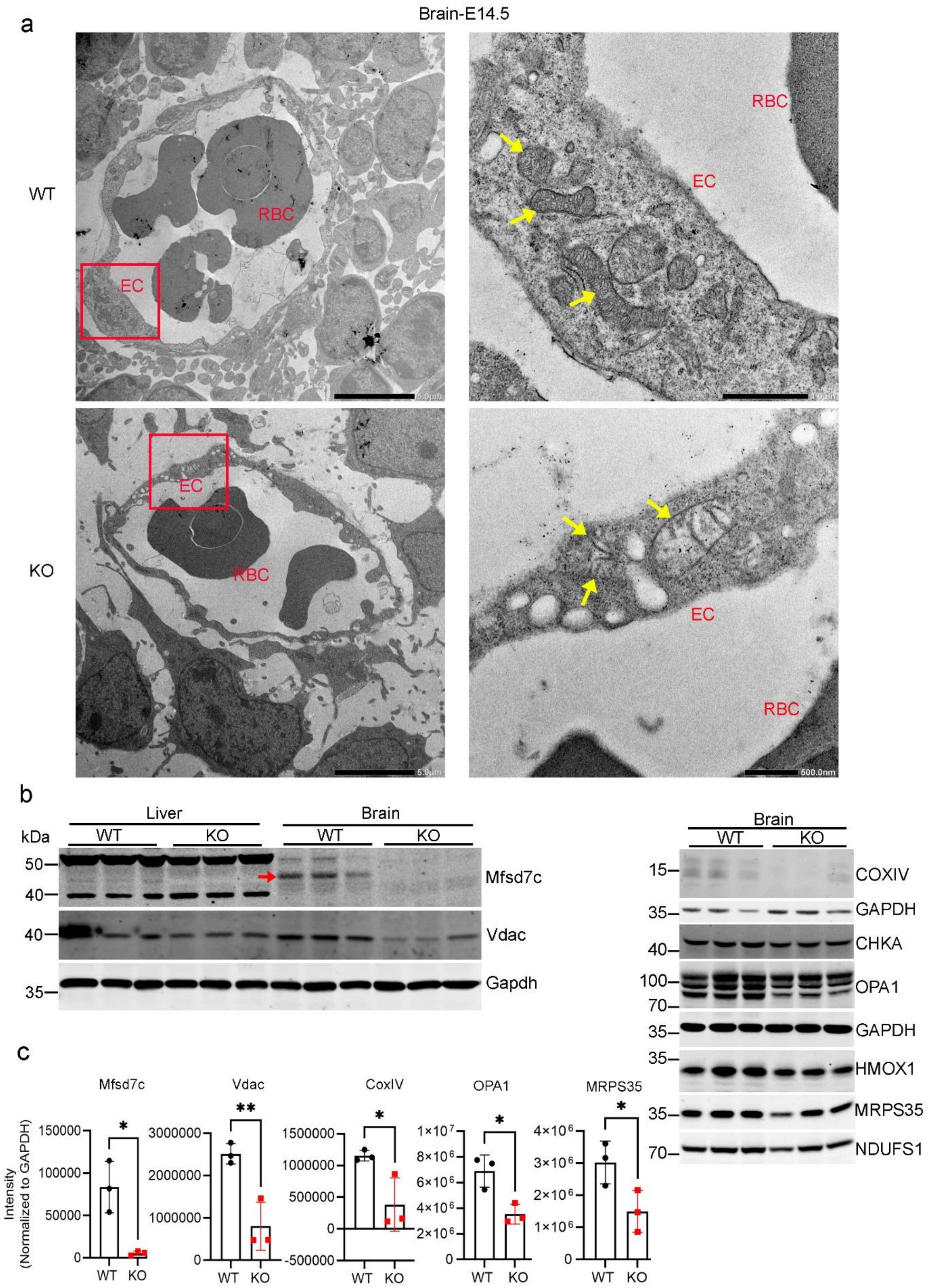

Supplementary information, Fig. S2. Mitochondrial defects in the brain of Mfsd7c knockout embryos. a, *Representative images of electron microscopic graphs of mitochondrial morphology of the CNS endothelial cells from E14.5 Mfsd7c KO and controls.*

Arrows show mitochondria which were enlarged in the Mfsd7c knockouts. n=3 per genotype. ***b****, Western blot analysis of Mfsd7c expression in the livers and brains as well as the mitochondrial markers (CoxIV, OPA1, MRPS35, VDAC, NDUFS1) cytosolic markers such as choline kinase A (CHKA) and heme oxygenase 1 (Hmox1) in the brains of E14.5 Mfsd7c KO and controls. Mfsd7c expression is present in the brain, not liver of the embryos.* ***c****, Quantification of the mitochondrial protein bands from the Western blot analysis shown in b. The levels of mitochondrial proteins were significantly reduced in the brains of E14.5 Mfsd7c KO compared to controls. Experiments were repeated twice in triplicate (n= 3 per genotype). **P<0.01, *P<0.05; ns, not significant. t-test was used.*
