# Supplementary material for: MFSD7c functions as a transporter of choline at the blood–brain barrier
Source: Cell Res. 2024 Feb 2;34(3):245–57. doi: 10.1038/s41422-023-00923-y (PMC10907603; doi:10.1038/s41422-023-00923-y)
Supplement: Supplementary file 4 — Supplementary information Fig S4 [file 41422_2023_923_MOESM4_ESM.pdf]

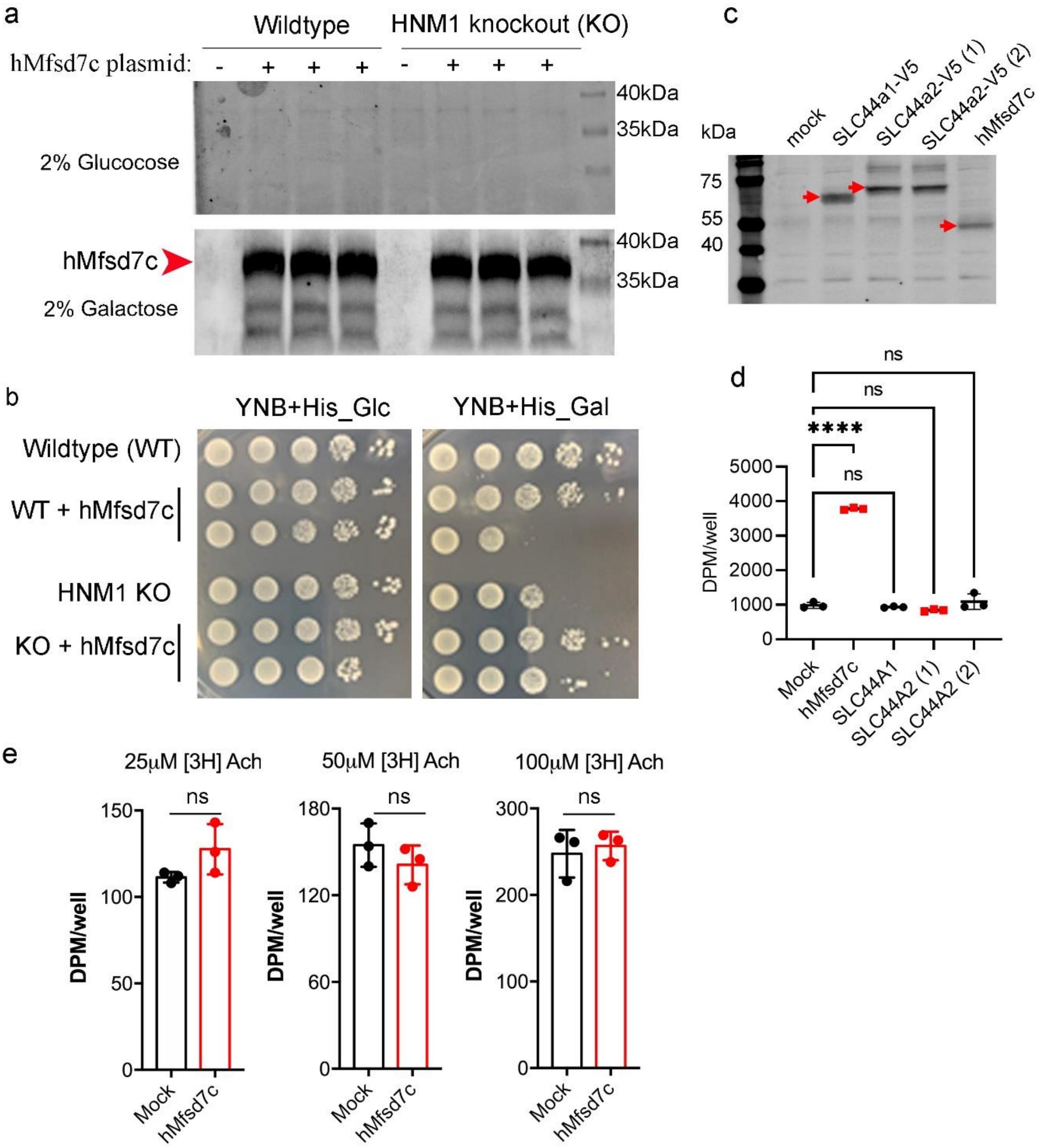

Supplementary information, Fig. S4. Human Mfsd7c functionally rescues for the loss of HNM1, the sole choline transporter in yeasts. ***a-b***, *Overexpression of human Mfsd7c in wild-type and HNM1 knockout yeast cells. Gal1 promoter was used for expression of hMfsd7c. Thus, hMfsd7c expression is repressed by glucose (Glc) and induced by galactose (Gal). Yeasts with overexpression of hMfsd7c grew normally in YNB medium with glucose or galactose. Note that 2-3 different transformants with overexpression of hMfsd7c were used. Experiments were repeated with reproducibility.* ***c-d.*** *human SLC44a1 and SLC44a2 did not exhibit choline import activity. Human SLC44a1-V5 and SLC44a2-V5 were used.* ***e***, *Mfsd7c does not transport acetylcholine. ****P<0.0001, One-way ANOVA was used in d; t-test was used in e.*
