# Supplementary material for: MFSD7c functions as a transporter of choline at the blood–brain barrier
Source: Cell Res. 2024 Feb 2;34(3):245–57. doi: 10.1038/s41422-023-00923-y (PMC10907603; doi:10.1038/s41422-023-00923-y)
Supplement: Supplementary file 5 — Supplementary information Fig S5 [file 41422_2023_923_MOESM5_ESM.pdf]

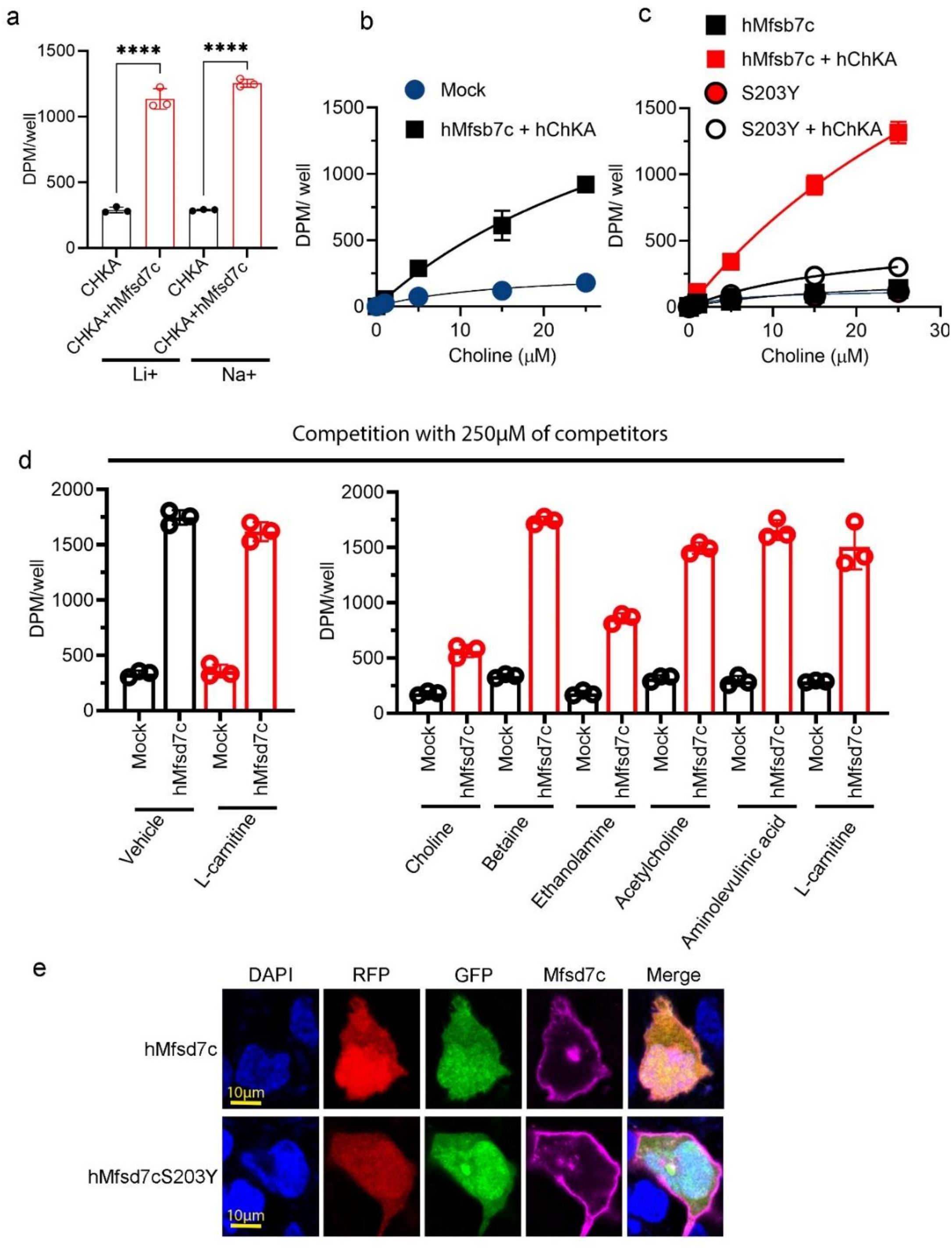

Supplementary information, Fig. S5. Choline transport properties of Mfsd7c, related to Figure 3. ***a***, *Mfsd7c activity is not sodium dependent. In these experiments, Mfsd7c was co-expressed with choline kinase A (hChKA). Transport assays were performed in transport buffer with lithium (Li+) in replacement of sodium (Na+). ****P<0.0001. One-way ANOVA was used.* ***b-c***, *Dose-curve of choline transport activity by hMfsd7c and S203Y mutant. Experiments were stopped after 30 mins of choline incubation. Experiments were performed*

in triplicate. ***d****, Competition assays of choline with indicated compounds. A 5-fold excess (250µM) of cold choline and ethanolamine, but not other indicated compounds reduced the import of radioactive choline [^{3}H]-choline. Experiments were repeated twice in triplicate. Data are expressed as mean ± SD.* ***e****, Representative images of co-expression of CHKA in RFP plasmid with hMfsd7c in GFP plasmid for single cell patch clamp. Cells with co-expression of the two fluorescent proteins were selected for patch clamp. Note that Mfsd7c (magenta) is localized in the plasma membrane.*
