# Supplementary material for: MFSD7c functions as a transporter of choline at the blood–brain barrier
Source: Cell Res. 2024 Feb 2;34(3):245–57. doi: 10.1038/s41422-023-00923-y (PMC10907603; doi:10.1038/s41422-023-00923-y)
Supplement: Supplementary file 6 — Supplementary information Fig S6 [file 41422_2023_923_MOESM6_ESM.pdf]

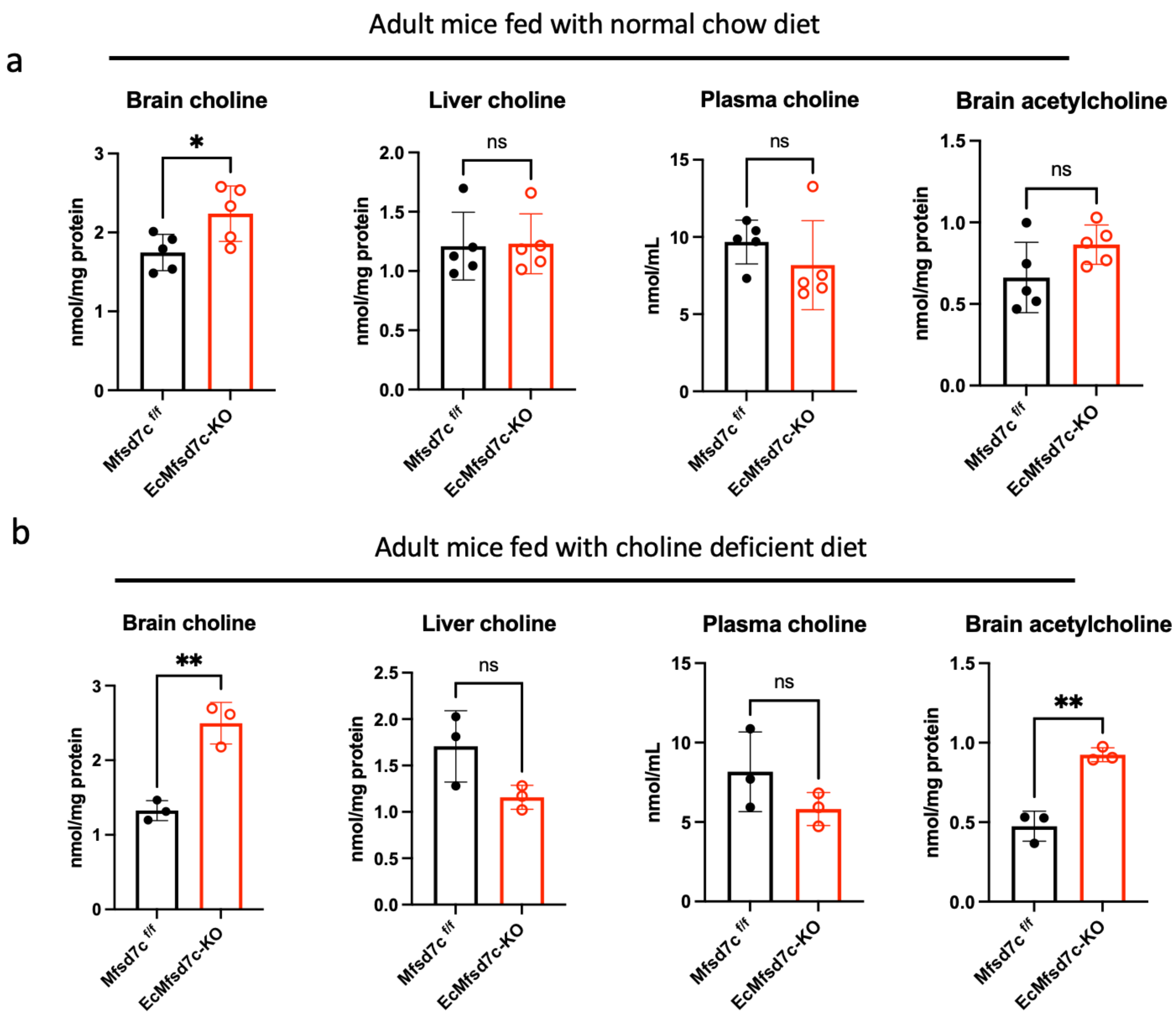

Supplementary information, Fig. S6. Increased levels of choline and acetylcholine in the brain of adult EcMfsd7c-KO mice. *a,* *choline and acetylcholine levels in the brain, liver, and plasma of EcMfsd7c-KO and control mice under normal chow diet.* ***b,*** *choline and acetylcholine levels in the brain, liver, and plasma of EcMfsd7c-KO and control mice after feeding for 1-1.5months with choline deficient diet. Each symbol represents one mouse. Data are expressed as mean ± SD. **P<0.001, *P<0.05; t-test was used.*
