# Supplementary material for: MFSD7c functions as a transporter of choline at the blood–brain barrier
Source: Cell Res. 2024 Feb 2;34(3):245–57. doi: 10.1038/s41422-023-00923-y (PMC10907603; doi:10.1038/s41422-023-00923-y)
Supplement: Supplementary file 7 — Supplementary information Fig S7 [file 41422_2023_923_MOESM7_ESM.pdf]

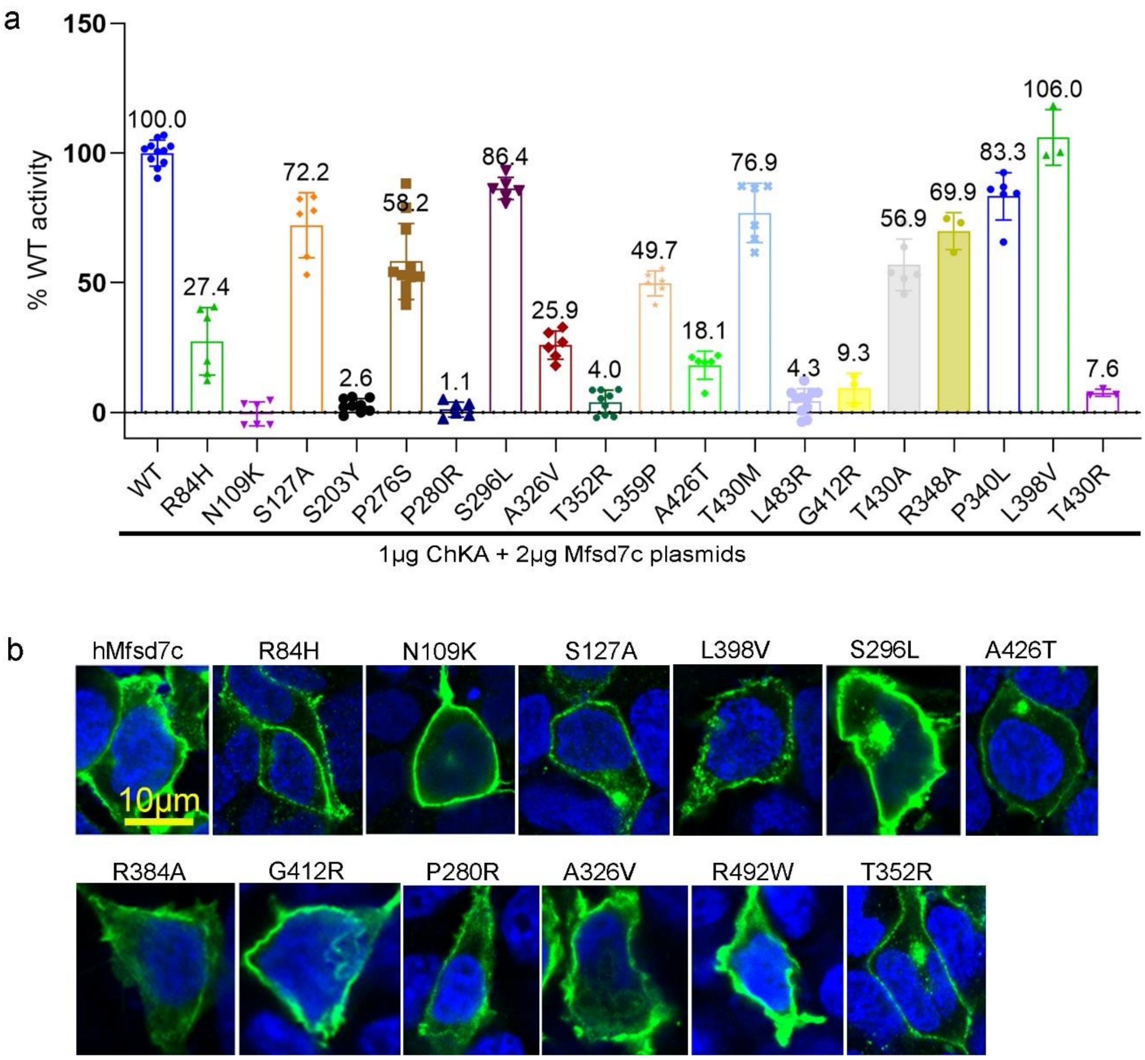

Supplementary information, Fig. S7. Missense mutations of Mfsd7c affect choline transport activity. a, *Transport activity of the missense mutations of Mfsd7c that were reported previously in the literature. Transport activity of these missense mutations was expressed as percentage of wildtype (WT) protein. Each symbol represents one replicate. Experiments were repeated at least twice.* ***b****, Fluorescent microscope analysis of the localization of the missense mutations in HEK293 cells. Localization of these missense mutations to the plasma membrane as similar to the wild-type protein was not affected. A full list of mutants can be found in the* ***Supplementary information, Table S13****.*
