# Supplementary material for: MFSD7c functions as a transporter of choline at the blood–brain barrier
Source: Cell Res. 2024 Feb 2;34(3):245–57. doi: 10.1038/s41422-023-00923-y (PMC10907603; doi:10.1038/s41422-023-00923-y)
Supplement: Supplementary file 8 — Supplementary information Fig S8 [file 41422_2023_923_MOESM8_ESM.pdf]

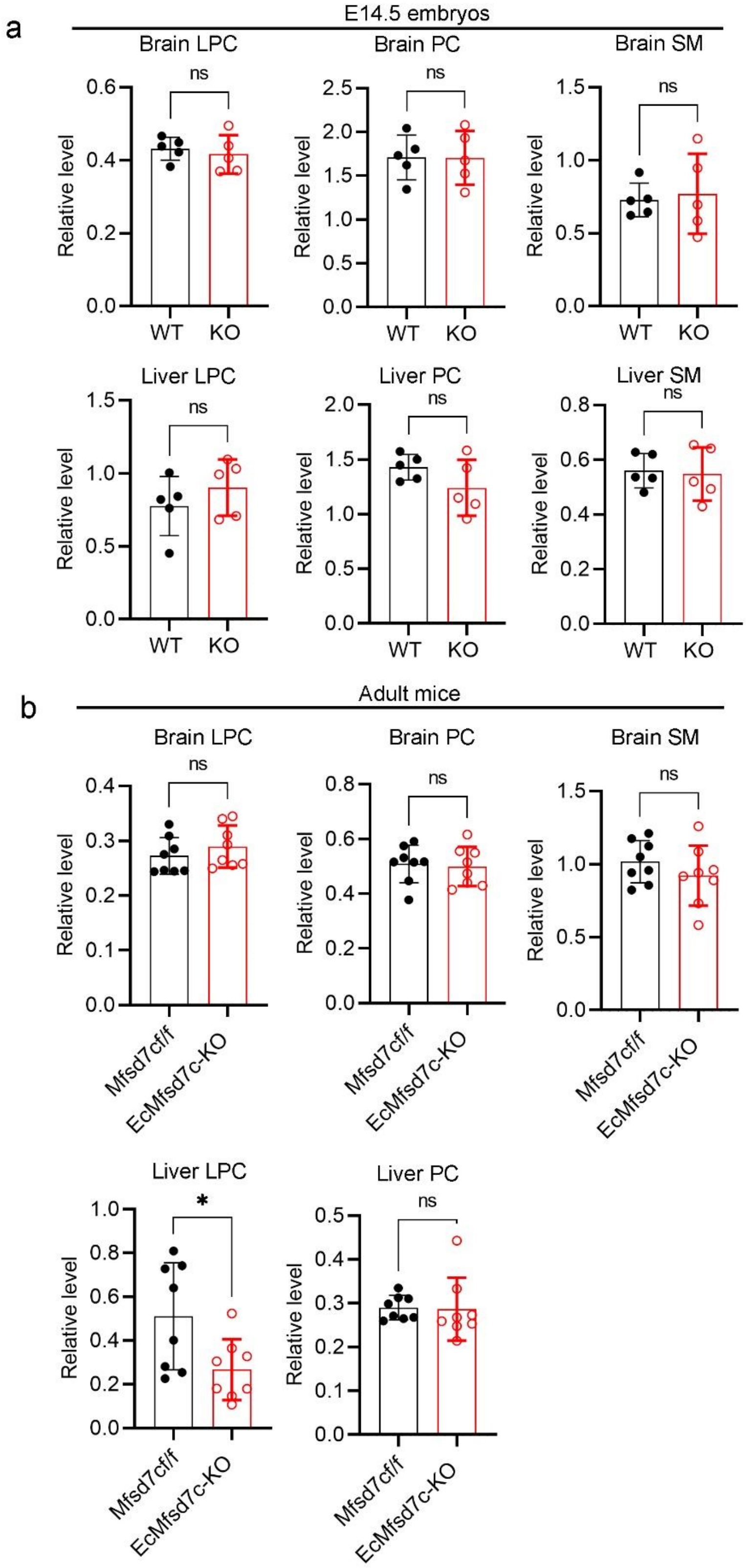

Supplementary information, Fig. S8. Deletion of Mfsd7c did not affect the levels of endogenous phospholipids in the brain and liver. a, *Lipidomic analysis of phospholipids containing choline including LPC, PC, and SM in the brains and livers of E14.5 Mfsd7c KO and wild-type littermates.* ***b,*** *Lipidomic analysis of LPC, PC, and SM in the brains and livers*

*from adult EcMfsd7c-KO and control mice. Each symbol represents one mouse. Data are expressed as mean ± SD. *P<0.05; t-test was used. Full list of phospholipids can be found in the* ***Supplementary information, Tables S14-17****.*
