# Supplementary material for: MFSD7c functions as a transporter of choline at the blood–brain barrier
Source: Cell Res. 2024 Feb 2;34(3):245–57. doi: 10.1038/s41422-023-00923-y (PMC10907603; doi:10.1038/s41422-023-00923-y)
Supplement: Supplementary file 9 — Supplementary information Fig S9 [file 41422_2023_923_MOESM9_ESM.pdf]

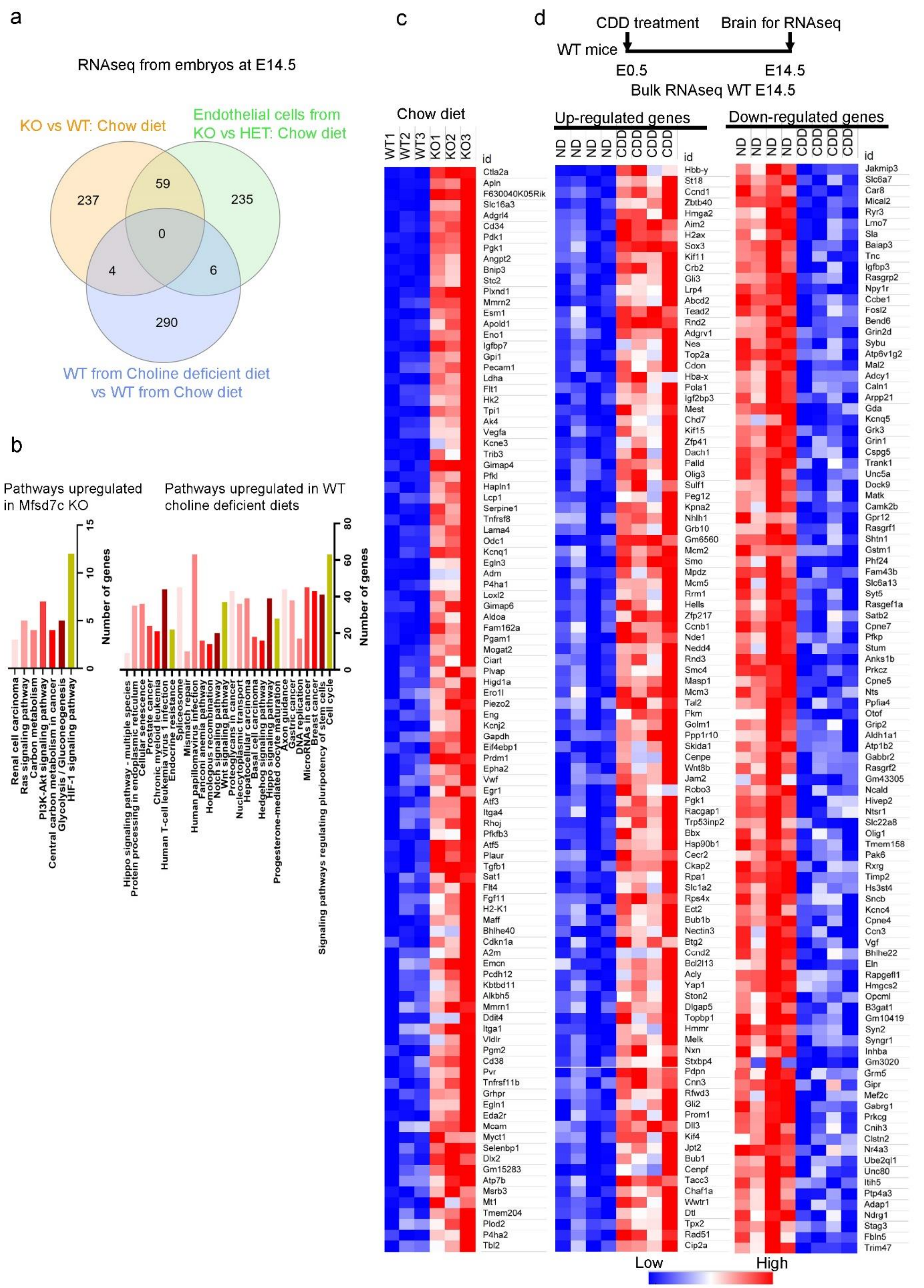

Supplementary information, Fig. S9. Gene expression changes due to maternal choline deficiency did not recapitulate the loss of Mfsd7c in the embryos. ***a,*** *Venn diagram of differential gene expression change in the brains of Mfsd7c knockout embryos and wildtype*

embryos from maternal choline deficiency. In comparison amongst the top 300 genes that were differentially changes in the whole brain and isolated CNS endothelial cells from Mfsd7c knockout, there were a significant overlap. However, there was a few genes that were changed due to choline deficiency compared to the lack of Mfsd7c. ***b****, Upregulated biological pathways in the brain of Mfsd7c KO embryos from mothers fed with choline sufficient diet and wild-type embryos from mothers fed with choline deficient diet.* ***c****, Heatmaps of upregulated genes from Mfsd7c KO compared to wild-type littermates.* ***d****, Heatmaps of upregulated and downregulated genes from wild-type embryos from mothers fed with choline deficient diet compared to wild-type embryos from mothers fed with choline sufficient diet. There is no major overlap of upregulated pathways due to Mfsd7c deletion and choline deficiency. The RNAseq dataset for WT from ND and CDD can be found in the* ***Supplementary information, Table S18****.*
