# Supplementary material for: MFSD7c functions as a transporter of choline at the blood–brain barrier
Source: Cell Res. 2024 Feb 2;34(3):245–57. doi: 10.1038/s41422-023-00923-y (PMC10907603; doi:10.1038/s41422-023-00923-y)
Supplement: Supplementary file 10 — Supplementary information Fig S10 [file 41422_2023_923_MOESM10_ESM.pdf]

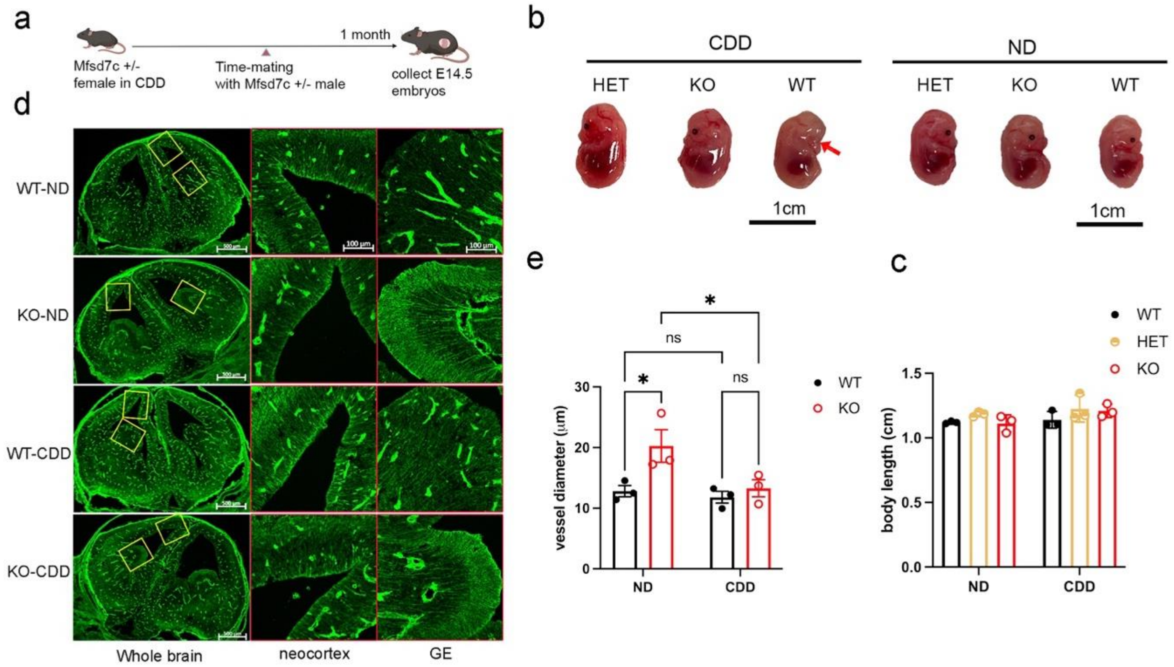

Supplementary information, Fig. S10. Defective choline export in the brain might confer to the phenotypes in the mice. *a,* *Illustration of treatment regimen for the mice with choline deficient diet (CDD).* ***b,*** *Maternal choline deficiency affects embryonic development. Arrow shows failed development of eyes in a WT embryo.* ***c****, Quantification of body length of WT, HET, and KO E14.5 embryos from normal chow diet (ND) and CDD diet. Each symbol represents one mouse.* ***d****, Depletion of choline ameliorated the dilation of the CNS blood vessels (marked by Glut1 expression) in the cortical regions but not in the ganglionic eminences (GE) of E14.5 KO embryos.* ***e****, Diameters of CNS blood vessels in the neocortex of KO embryos were decreased after CDD treatment. Note that there was no improvement in the phenotypes of the blood vessels in the ganglionic eminence (GE) regions. Data are expressed as mean ± SEM. Each symbol represents the averaged diameters of blood vessels collected from different images from 1 embryo. n=3 embryos per genotype. *P<0.05, two-way ANOVA*
