# Supplementary material for: MFSD7c functions as a transporter of choline at the blood–brain barrier
Source: Cell Res. 2024 Feb 2;34(3):245–57. doi: 10.1038/s41422-023-00923-y (PMC10907603; doi:10.1038/s41422-023-00923-y)
Supplement: Supplementary file 3 — Supplementary information Fig S3 [file 41422_2023_923_MOESM3_ESM.pdf]

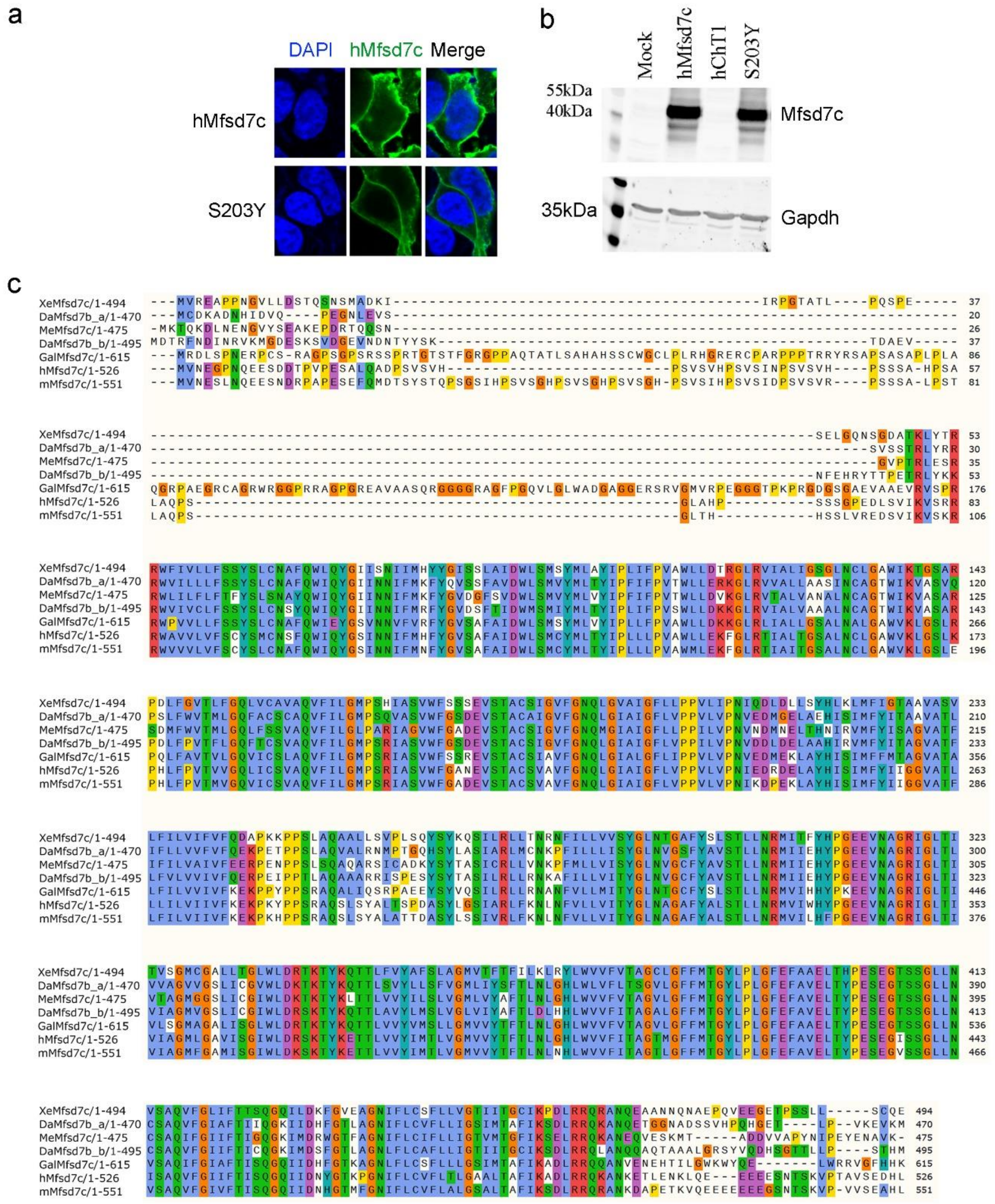

Supplementary information, Fig. S3. Mfsd7c is localized in the plasma membrane and conserved from fish to man, related to Figure 2. ***a-b,*** *Expression of hMfsd7c and S203Y mutant in HEK293 cells. hChT1 is neuronal choline transporter was used as negative control for Western blot analysis.* ***c****, Alignment of human (hMfsd7c), frog (XeMfsd7c), Zebrafish (DaMfsd7c_a and DaMfsd7c_b isoforms), Medaka fish (mMfsd7c), Chicken (GaMfsd7c), and mouse (mMfsd7c) Mfsd7c orthologs. Mfsd7c is not conserved in yeasts.*
